# Anticancer potential of 2,2′-bipyridine hydroxamic acid derivatives in head and neck cancer therapy

**DOI:** 10.1007/s10822-025-00640-1

**Published:** 2025-08-06

**Authors:** Manasa Gangadhar Shetty, Bipasa Dey, Padmini Pai, Babitha Kampa Sundara, Kapaettu Satyamoorthy, Srinivas Oruganti, Usha Yogendra Nayak, T. Ashwini

**Affiliations:** 1 Department of Biophysics, Manipal School of Life Sciences, Manipal Academy of Higher Education, Manipal, Karnataka 576104 India; 2 Shri Dharmasthala Manjunatheshwara (SDM) University, Manjushree Nagar, Sattur, Dharwad, Karnataka 580009 India; 3 Dr. Reddy’s Institute of Life Sciences, University of Hyderabad Campus, Gachibowli, Hyderabad, 500046 India; 4 Department of Pharmaceutics, Manipal College of Pharmaceutical Sciences, Manipal Academy of Higher Education, Manipal, Karnataka 576104 India

**Keywords:** Anticancer, Head and neck cancer, Histone deacetylases, Hydroxyurea

## Abstract

The genesis of head and neck cancer (HNC) is attributed to the combined influence of genetic and epigenetic irregularities. While surgical resection and radiotherapy remain primary treatment modalities, the effectiveness of current chemotherapeutic options is often hindered by toxicity, resistance, and limited selectivity. Hydroxyurea has long been recognized for its anticancer potential; however, its clinical application is limited by a short half-life, dose-dependent toxicity, and resistance mechanisms. To address these limitations, researchers have focused on developing novel hydroxyurea derivatives with improved pharmacokinetics, target specificity, and multimodal mechanisms of action. In the present study, we report the design and synthesis of two novel 2,2′-bipyridine hydroxamic acid derivatives, including a hydroxyurea analogue aimed at enhancing chemotherapeutic efficacy and safety. Compound **1A** demonstrated selective cytotoxicity against Cal27 cells (IC_50_ = 19.36 μM). Mechanistic investigations revealed that **1A** inhibits cancer cell migration and induces ROS-mediated apoptosis. Additionally, **1A** exhibited moderate HDAC inhibition, supported by molecular docking and dynamics simulations, which confirmed stable binding to HDAC 2 isoform through Zn^2^⁺ coordination. These findings place compound **1A** as a promising lead candidate, integrating epigenetic modulation and direct cytotoxic effects for potential therapeutic application in HNC.

**Supplementary Information:**

The online version contains supplementary material available at 10.1007/s10822-025-00640-1.

## Introduction

**Fig. 1:**
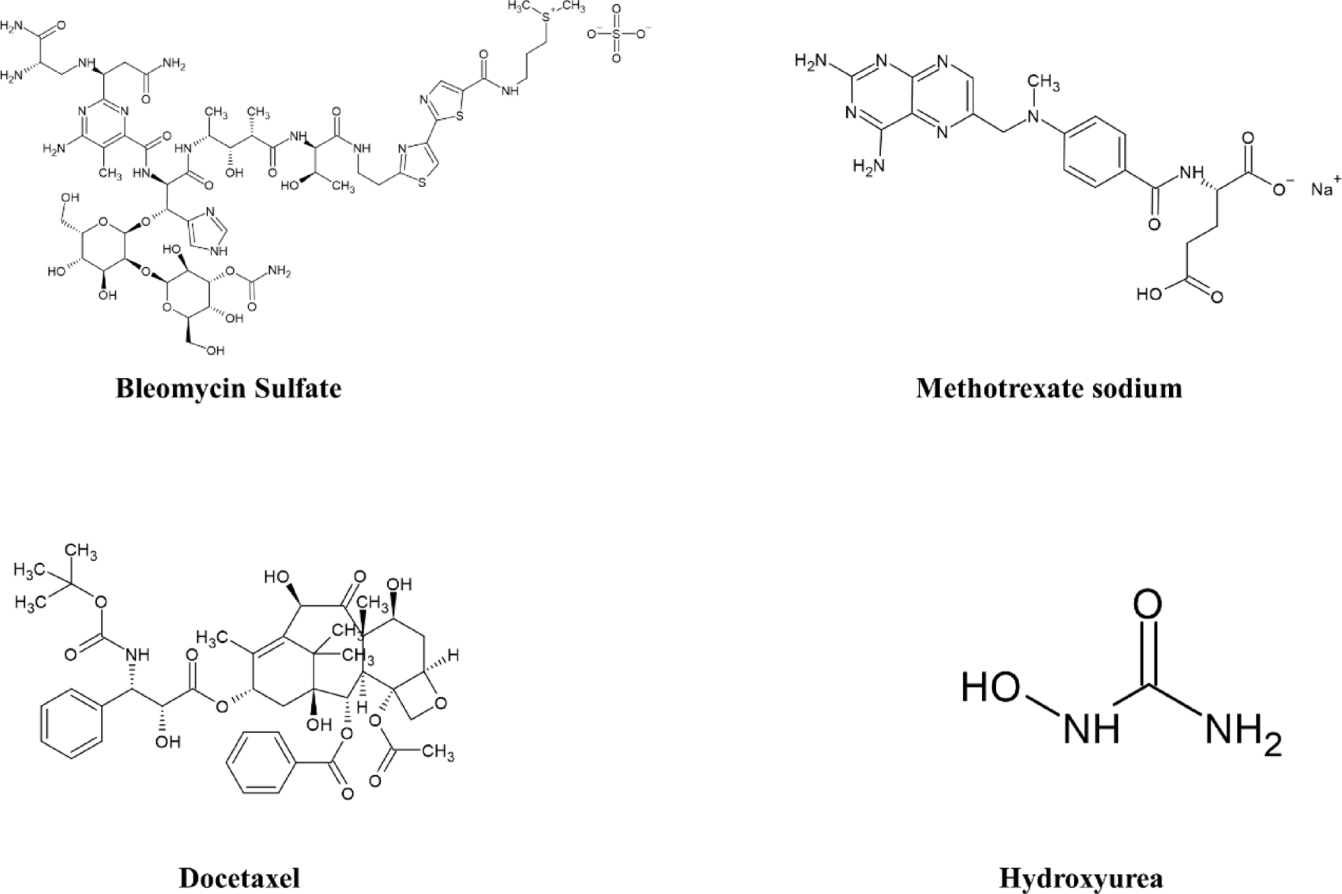
Chemical structures of drugs used in the treatment of HNC

Head and neck cancer (HNC) is the seventh most prevalent type of cancer globally with over 660,000 new cases and 325,000 annual mortalities. Oral squamous cell carcinoma (OSCC), constituting approximately 90% of HNCs, originates from the epithelial lining of the oral cavity, pharynx, and larynx. In 2020, it was reported that 476,125 people were diagnosed with oral or oropharyngeal cancer, highlighting the significant ill impact of this disease on human population globally [1]. OSCC is known for its invasive and aggressive characteristics, along with early metastasis and significant lymph node involvement [2]. Despite advancements in cancer treatment, OSCC still faces considerable limitations in the available therapeutic approaches such as surgery, chemotherapy, and radiotherapy [3]. The inadequate robust treatment choices result in elevated morbidity, diminished quality of life, and a heightened incidence of metastatic disease [4]. Consequently, there is a great need for the development of potential therapeutic strategies to address these challenges. To date, only few drugs have been used for the treatment of cancers that arises in the head or neck region. These drugs include chemotherapeutic agents, targeted therapies, and immune checkpoint inhibitors. Table 1 summarizes their names; mechanisms of action and Food and Drug Administration (FDA) approval status and Fig. 1 represents their chemical structures.

**Table 1:** Summary of drugs used in the treatment of HNC [5]

Generic name	Brand name(s)	Drug class	Key use in HNC
Cetuximab	Erbitux	Anti-EGFR monoclonal antibody	Locally advanced/metastatic HNC (with radiotherapy or alone)
Nivolumab	Opdivo	Anti-PD-1 monoclonal antibody	Recurrent/metastatic HNC post-platinum therapy
Pembrolizumab	Keytruda		Recurrent/metastatic HNC (PD-L1^+^ or platinum-refractory)
Toripalimab-tpzi	Loqtorzi		Recurrent/metastatic nasopharyngeal carcinoma (NPC)
Penpulimab-kcqx	Ak104		Recurrent/metastatic nasopharyngeal carcinoma
Bleomycin sulfate	Blenoxane	DNA strand breaker	Off-label: Palliative or combination regimens
Docetaxel	Taxotere	Taxane (Microtubule inhibitor)	
Hydroxyurea	Droxia Hydrea	Ribonucleotide reductase inhibitor	Off-label: Combined with radiation (historic use)
Methotrexate sodium	Trexall Xatmep	Antimetabolite (DHFR inhibitor)	Off-label: Palliative therapy

2,2′-bipyridine is an important bidentate ligand that co-ordinates with metal ions to form stable metal complexes. These complexes have shown great promise in various biomedical applications due to their unique chemical properties. In targeted anticancer therapies, metal complexes of 2,2′-bipyridine are particularly interesting because they can engage in selective binding with biomolecules, offering controlled drug release and potential cytotoxic effects against cancer cells.

The coordination of 2,2′-bipyridine with metals like platinum, ruthenium, and copper has been studied extensively for anticancer purposes [6].

HU has been recognized as a valuable anticancer compound, exhibiting efficacy in the treatment of various malignancies including HNC as shown in Table 1. However, the short life of HU necessitates the use of higher doses causing toxicity, and the emergence of resistance in many cancer types. The development of resistance poses a significant challenge in the clinical management of cancer, limiting the effectiveness of HU as a therapeutic agent [7, 8]. This has emphasized the need for the development of novel HU derivatives to enhance and diversify anticancer potential.

Researchers are actively engaged in the synthesis of novel HU derivatives as a strategic approach to enhance the compound's anticancer activity and overcome resistance mechanisms [8-11]. By tailoring the chemical structure of HU, researchers aim to develop derivatives with enhanced bioavailability, improved target specificity, and a broader spectrum of anticancer effects. Studies have demonstrated promising results with novel HU derivatives, exhibiting their potential to circumvent resistance mechanisms observed in traditional HU treatments.

**Fig. 2:**
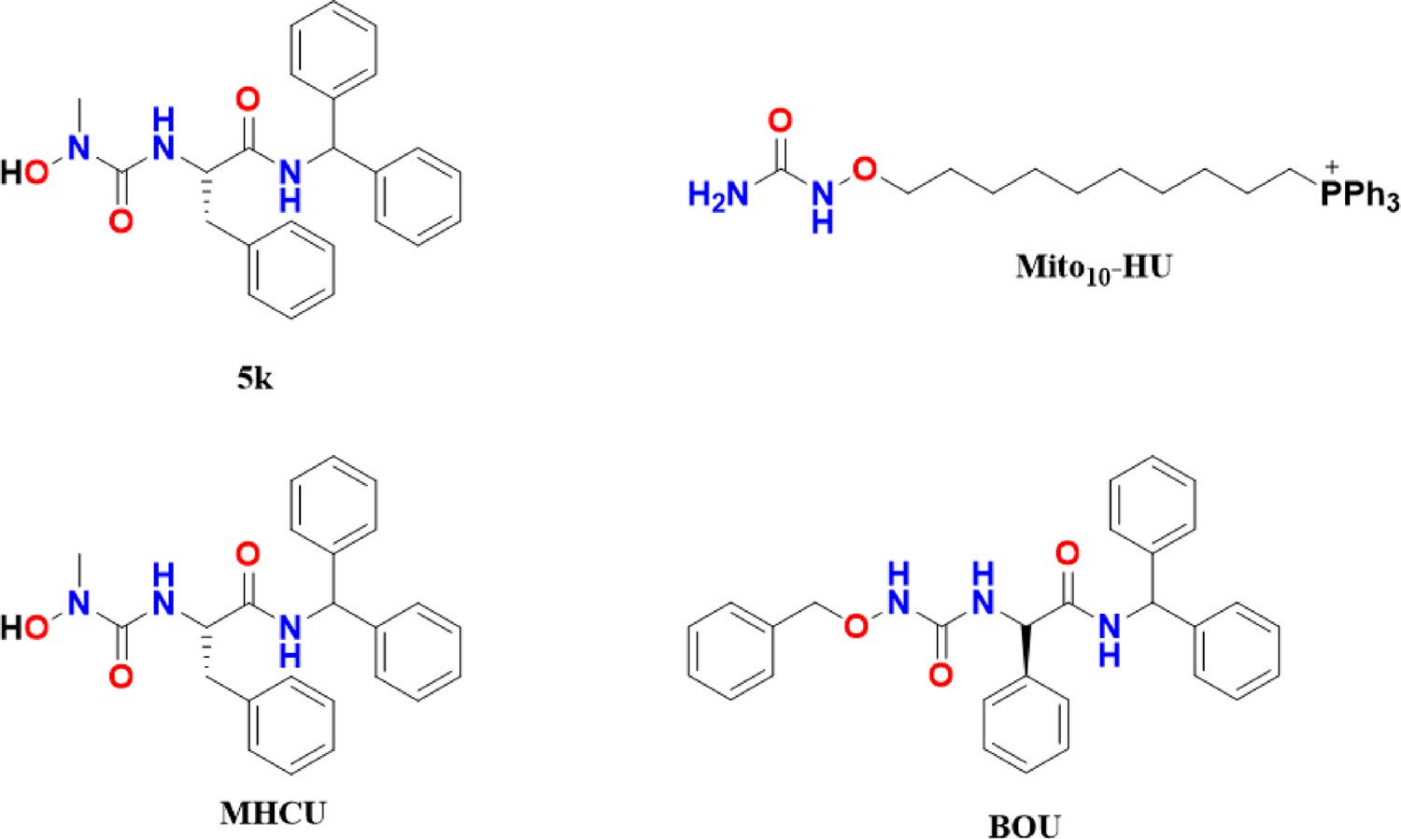
Chemical structures of novel HU derivatives developed as anticancer agents

A series of novel lipophilic L- and D-amino acid HU derivatives (5a–l) were synthesized and tested for antiviral and anticancer activity by a Perkovic et al., in 2008. Among them, compounds 5a, 5c, 5e, and 5 k showed the most potent antiproliferative effects across various tumor cell lines. These compounds also demonstrated significant, non-specific cytostatic effects in cervical (HeLa), breast (MCF-7), pancreatic (MIA PaCa-2), liver (HepG2), prostate (PC-3), and colorectal (SW620) cancer cells, especially at micromolar concentrations, indicating promising potential as broad-spectrum anticancer agents [8]. Further in 2013, Saban et al., developed two novel HU derivatives, namely, BOU and MHCU, which exhibited similar antiproliferative effects but distinct mechanisms of action, including differential HDAC class I/II inhibition. These differences resulted from their structural variations, BOU contains a benzyl group, while MHCU has a free hydroxyl group. BOU demonstrated slightly higher potency against colorectal cancer SW620 cells, inducing apoptosis through oxidative stress and DNA damage. In contrast, MHCU primarily changed anti-inflammatory pathways. BOU showed significant short-term antitumor effects in vivo, linked to histone deacetylases (HDAC) inhibition, oxidative stress, and inflammation modulation [9]. Cheng et al., in 2021 modified HU by replacing its hydroxyl group with a triphenylphosphonium cation linked to alkyl chains of varying lengths, creating mitochondria-targeted derivatives (Mito-HUs). Increasing the alkyl chain length enhanced hydrophobicity, boosting their ability to inhibit oxidative phosphorylation and tumor cell proliferation. These Mito-HUs also suppressed mitochondrial complexes I and III, reducing oxygen consumption. The more hydrophobic variants further blocked immunosuppressive myeloid cells while activating T cells, suggesting potential immunomodulatory antitumor effects [10] (Fig. 2).

Furthermore, due to the presence of hydroxamic acid moieties, well-known zinc-binding group of HDAC inhibitors in the developed compounds, the compounds are also evaluated for their HDAC inhibitory effect. HDACs are key epigenetic regulators that remove acetyl groups from histone and few non-histone proteins, leading to chromatin condensation and transcriptional repression. Aberrant expression of HDAC activity has been associated in the silencing of tumor suppressor genes, promoting uncontrolled cell proliferation and cancer growth in several cancers. In particular, the overexpression of HDACs 1 and 6 in HNC correlates with poor prognosis and cancer stem cell maintenance. Thus, HDACs have emerged as promising therapeutic targets for cancer therapy including HNCs [12]. The present work reports the design and synthesis of two novel 2,2′-bipyridine derivatives and their biological evaluation for anticancer activity, targeting epigenetic pathways relevant to HNC progression.

## Methodology

### Pharmacology analysis, synthetic accessibility, and novelty of the developed compound prediction

Following the design of compounds, **1A** and **1B**, SciFinder was employed to predict the novelty of the two compounds. The pharmacological and pharmacokinetic properties were evaluated according to Lipinski's rule of 5, encompassing an ADMET (Absorption, Distribution, Metabolism, Excretion, and Toxicity) test conducted through *in-silico* analysis using the SwissADME and pkCSM online tools. In addition, the SwissADME web interface was used to assess the synthetic accessibility of the compounds [13–15].

### Chemistry

#### General information

The starting materials and catalysts used in the chemical synthesis, as well as the chemicals for buffer preparations, antibodies, and fluorescent dyes in this study, were purchased from various suppliers. All commercially available reagents were used as received. Reaction progress was tracked by analytical thin layer chromatography (TLC) on silica gel plates, visualized under ultraviolet (UV) light at 254 and 366 nm. Compound purities were evaluated using high-performance liquid chromatography (HPLC) with a Waters Alliance system and Shimadzu equipment using a suitable mobile phase on a Waters C18 column, and through melting point determination with a digital capillary apparatus. High-resolution mass spectra (HRMS) were obtained with an Agilent 6520 Q-TOF. Fourier-transform infrared (FTIR) spectra were recorded with a Shimadzu FTIR-8310, and ^1^H and ^13^C nuclear magnetic resonance (NMR) spectra were acquired using a Bruker Ascend instrument.

#### Synthesis and characterization of N-hydroxy-2,2′-bipyridine-6-carboxamide (1A)

5.5 mL of 30% hydrogen peroxide was added to 5 g (32 mmol) of 2,2′-bipyridine in 25 mL of trifluoroacetic acid. The reaction was stirred for 2 h followed by neutralization using 6 N sodium hydroxide and extracted with chloroform (3 × 150 ml) to obtain 2,2′-bipyridine-*N*-oxide. Subsequently, the reaction mixture of 3 g (17 mmol) of 2,2′-bipyridine-*N*-oxide in 30 mL of dichloromethane under nitrogen atmosphere was cooled to 0 °C and 8.72 mL (69 mmol) of trimethylsilyl cyanide and 4.08 mL of benzoyl chloride (34 mmol) were added to the reaction mixture, which was then maintained at room temperature with continuous stirring for 16 h to yield 2,2′-bipyridine-6-carbonitrile. 500 mg (2.7 mmol) of 2,2′-bipyridine-6-carbonitrile was then added with 10 mL of ethanol and 25 mL of sulfuric acid and the mixture was heated to 70–80 °C and stirred for 36 h to yield ethyl 2,2′-bipyridine-6-carboxylate. Finally, 0.4 g (1.7 mmol) of ethyl 2,2′-bipyridine-6-carboxylate, 0.182 g (2.6 mmol) of hydroxylamine hydrochloride, and 0.628 mL (3.5 mmol) of *N,N*′*-*diisopropylethylamine were combined at room temperature. The reaction mixture was heated to 75 °C and stirred for 48 h and the resulting crude product was purified by column chromatography and the product was eluted with 5–7% of methanol/dichloromethane.

White solid; mp: 107–112 °C; Purity 99.59% (HPLC); Yield: 33%; FTIR (KBr, cm^−1^): 3223.05 (NH, OH); 1664.57 (CO); 1573.91 (NH); 1257.59 (CN). ^1^H NMR (400 MHz, DMSO-d6, ppm): δ 11.70 (s, 1H, hydroxamic NH); 9.26 (s, 1H, hydroxamic OH); 8.94 (d, 1H, *J* = *7.6 Hz*); 8.71 (d, 1H*, J* = *4.4 Hz*); 8.56 (d, 1H, *J* = *7.6 Hz*); 8.12 (t, 1H, *J* = *8.0 Hz*); 8.04 (d, 1H, *J* = *7.2 Hz*); 7.98 (t, 1H, *J* = *8.0 Hz*); 7.49 (t, 1H, *J* = *5.8 Hz*). ^13^C NMR (100 MHz, DMSO-d6, ppm): δ161.53; 154.86; 154.80; 150.03; 149.61; 139.25; 137.73; 125.09; 123.08; 122.39; 122.24. HRMS (Q-TOF) m/z calculated for C_11_H_9_N_3_O_2_, [M+H]^+^ 216.0768 g/mol. Found, 216.0334 g/mol. The spectral data of intermediates and compound **1A** is given in the supplementary file.

#### Synthesis and characterization of 1-([2,2′-bipyridin]-6-yl)-3-hydroxyurea (1B)

4 g (23 mmol) of 2,2′-bipyridine-*N*-oxide was dissolved in 40 mL of 2,2,2-trifluoro toluene. The reaction mixture was then cooled to 0 °C, followed by the sequential addition of 12.2 mL (11.6 mmol) of tertiary butylamine and 13.64 g (69 mmol) of p-toluenesulfonyl chloride. After stirring for 18 h, 200 mL of trifluoroacetic acid was added, and the mixture was heated to 90 °C for 24 h. Trifluoroacetic acid was then removed, and the resulting compound was acidified with 6 N hydrochloric acid (pH 2–3) and then washed with methyl tertiary butyl ether: ethyl acetate (50 mL: 50 mL). The aqueous layers were then basified with 6 N sodium hydroxide (pH 10–12) to get (2,2′-bipyridin)-6-amine.

In a separate reaction, 1.21 g (7.6 mmol) of *O*-benzyl hydroxylamine hydrochloride was added to a solution of 1.23 g (7.6 mmol) of 1,1′-carbonyldiimidazole and 1.3 g (7.6 mmol) of *N,N*′-diisopropylethylamine in dry tetrahydrofuran at room temperature under nitrogen (N_2_) atmosphere. After stirring for 30 min, this mixture was added to a solution of 650 mg (3.801 mmol) of (2,2′-bipyridin)-6-amine in 10 mL of tetrahydrofuran at room temperature under N_2_ atmosphere to 1-([2,2′-bipyridin]-6-yl)-3-(benzyloxy)urea. This compound was then subjected to hydrogenation with ethanol and palladium on carbon under a hydrogen pressure (H_2_) of 600 psi for 5 h. After completion of the reaction, the mixture was filtered over a celite bed and washed with 50 mL of ethanol. The crude product was then purified by column chromatography and eluted in 30–40% ethyl acetate-hexane.

White solid; mp: 167–173 °C; Purity 95.93% (HPLC); Yield: 45%; FTIR (KBr, cm^−1^): 3209.19 (NH, OH); 1657.89 (CO); 1555.75 (NH) 1244.53 (CN). ^1^H NMR (400 MHz, DMSO-d6, ppm): δ 9.46 (s, 1H, hydroxamic OH); 9.26 (s, 1H, amide NH); 8.76 (s, 1H, hydroxamic NH); 8.68 (d, 1H, *J* = *4.4 Hz*); 8.31 (d, 1H, *J* = *8.0 Hz*); 8.02 (d, 1H, *J* = *8.0 Hz*); 7.93 (m, 3H) and 7.45 (t, 1H, *J* = *6.2 Hz*), ^13^C NMR (100 MHz, DMSO-d6, ppm): δ157.66; 155.20; 154.02; 152.03; 149.76; 139.79; 137.75; 124.67; 120.90; 115.35; 112.82. HRMS (Q-TOF) m/z calculated for C_11_H_10_N_4_O_2_, [M + H]^+^ 231.0877 g/mol. Found, 231.0396 g/mol. The spectral data of intermediates and compound **1B** is given in the supplementary file.

### Biological evaluation

#### Cell culture

The human cancer cells, namely, SiHa (cervical), MCF7 (breast) and Cal27 (oral) were procured from the ATCC (Manassas, VA, USA). The normal human foreskin fibroblasts were isolated after obtaining the ethical clearance from the institutional ethics committee, Kasturba Medical College, MAHE, Manipal, India. Isolation of fibroblasts was performed using skin epidermis (human dermal fibroblasts (HDF). The cells were cultured in DMEM (Dulbecco's Modified Eagle Medium) with 10% FBS (Fetal Bovine Serum) (Himedia, India). The culture medium was then renewed every 48–72 h. The cells were cultured under optimum conditions by maintaining the cells in incubator (Eppendorf CellXpert® C170, Hamburg, Germany) with 5% carbon dioxide (CO_2_) and humidified air at 37 °C.

#### In-vitro cell viability assay

The cells were trypsinized using 0.25% trypsin, and their total count was determined using a haemocytometer. Subsequently, 10,000 cells/well for SiHa, Cal27, and MCF7 cells and 5000 cells/well for fibroblast cells were seeded in 96 well plate and incubated for 24 h in CO_2_ incubator at 37 °C. Then 5 mg/mL of 3[-dimethylthiazol-2-yl]-2,5-diphenyl-tetrazolium bromide (MTT) was added after a 48 h incubation with varying concentrations of the test compounds. Post MTT treatment, the plates were incubated for an additional 4 h at 37 °C. Subsequently, dimethyl sulfoxide (DMSO) was introduced to each well to dissolve the formazan crystals. The optical densities (O.D) of the samples were measured at 570 nm and 640 nm using Tecan Spark (Tecan, Männedorf, Switzerland). The results were represented as a percentage of the control and were computed as inhibitory concentration (IC_50_) values utilizing GraphPad Prism 9 software. Each compound was put in triplicate, and each experiment was replicated thrice [16].

#### In-vitro wound healing assay

2 × 10^5^ cells/well of Cal27 cells were seeded in 6 well plate to establish a monolayer. An artificial scratch was generated in the monolayer with a sterile 200 μL pipette tip which was followed by phosphate buffered saline (PBS) wash. Fresh culture medium, both without (control) and with the addition of test compounds, was then applied and incubated for 48 h. The closure of the scratch was observed and documented at 0, 24, and 48 h intervals using an inverted microscope (Zeiss, Neu-Isenburg, Germany). Scratch image analysis was conducted using ImageJ software (Wayne Rasband, USA). The progression of the open wound area percentage was graphically represented over time for each respective group.

#### Cell cycle analysis

1 × 10^6^ cells/mL of Cal27 cells were seeded in 10 cm plate and incubated for 24 h. They were then synchronized through serum starvation for 24 h, followed by treatment with compounds at their respective IC_50_ values for 48 h in culture medium. Afterward, cells were collected via trypsinization (0.25% trypsin), fixed overnight in 70% ethanol at 4 °C, treated with RNase A (10 µg/mL), and stained with propidium iodide (50 μg/mL) for 30 min at room temperature in the dark. Cell cycle distribution was analyzed using a Partec CyFlow Space with FloMax software (Partec, United States of America) [17].

#### Apoptosis assay

1 × 10^6^ cells/mL of Cal27 cells were seeded in 10 cm culture plates and allowed to grow till 70–75% confluency. They were then treated with compounds at their respective IC_50_ concentrations for 24 h. After treatment, the cells were washed twice with ice-cold PBS and incubated in the dark at room temperature in 100 mL of 1X binding buffer containing 1 µL Annexin V-fluorescein isothiocyanate (FITC) and 12.5 µL propidium iodide (PI). Following 15 min incubation, the percentage of apoptotic cells was analyzed using a Partec CyFlow Space with FloMax software (Partec, United States of America) [18].

#### Intracellular reactive oxygen species (ROS) measurement

1 × 10^6^ cells/mL of Cal27 were seeded in 10 cm culture plates and allowed to grow for 24 h until they reached 70–75% confluency. They were then treated with compounds at their respective IC_50_ concentrations for 48 h. Followed by treatment with 1 µg/mL diacetyldichlorofluorescein (H_2_DCFDA) dye for 45 min at 37 °C. Fluorescence of oxidized dichlorofluorescein (DCF) was measured using a Partec CyFlow Space with FloMax software (Partec, United States of America) [19].

#### Anchorage dependent colony formation assay

Cells were seeded at a density of 500 cells per well in 6-well culture plates. After 24 h of incubation, cells were treated with the compounds at their respective IC_50_ concentrations and incubated for 2 weeks with a continuously changing medium every three days. On the 14th day, the culture media was removed, colonies were stained with 0.4% crystal violet for 10 min, then followed by a PBS wash twice until the excess stain was removed, and the stained colonies were counted.

#### HDAC activity assay

HDAC fluorometric activity assay was employed to measure in vitro HDAC inhibition. Enzymes (HDAC), test compounds (inhibitors), and substrates were all diluted using HDAC assay buffer. In brief, HDAC assay buffer was added into each well, followed by the addition of test compounds (10 μL) to the assay buffer at various concentrations. Subsequently, a nuclear extract containing HDACs was added, and the mixture was incubated for 30 min. The enzymatic reaction was initiated by introducing fluorogenic substrates Boc-Lys(Ac)-AMC (MAL). After 2 h of incubation under shaking conditions at 37 °C, the reactions were halted by adding a stop solution (10 mg/mL trypsin and 0.2 mM Trichostatin A (TSA) in assay buffer). Following an additional 15 min of incubation at 37 °C, fluorescence was measured at emission wavelength of 354 nm and excitation wavelength 450 nm using a Tecan Spark (Tecan, Männedorf, Switzerland). The fluorescence in wells without test compounds was considered as control with 100% enzymatic activity [20].

### Molecular docking

The free binding energy (kcal/mol) for compound **1A** and the standard suberoylanilide hydroxamic acid (SAHA) to various HDAC isoforms was determined, using Maestro, Schrödinger (Version—11.2) as mentioned by Pai et al., 2022 [21].

### Molecular dynamic simulation

Molecular Dynamic (MD) simulation was conducted for compound **1A** and the standard SAHA in complex with HDAC 2 protein to enhance our insights into the stability of their interactions. The Desmond module of Schrodinger was employed for the MD simulations, following a three-step workflow as mentioned by Pai et al., in 2022 [21].

## Results and discussion

### Chemistry

#### Chemical synthesis

**Scheme 1:**
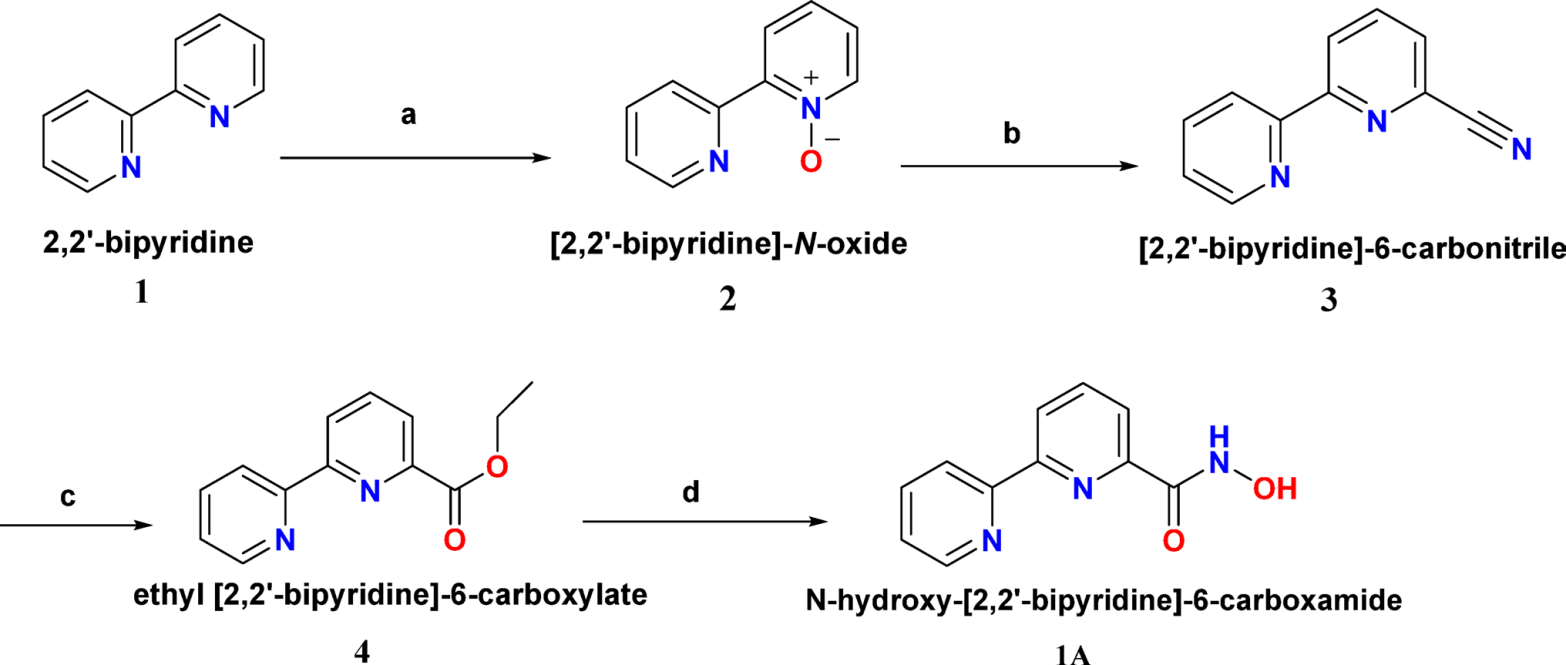
Chemical synthesis of compound **1A**, **a** trifluoroacetic acid and 30% hydrogen peroxide, room temperature, 2 h; **b** dichloromethane, N_2_, trimethylsilyl cyanide and benzoyl chloride, 16 h; **c** ethanol and sulphuric acid; **d** hydroxylamine hydrochloride, *N,N*′-diisopropylethylamine

The synthesis of compound **1A** involves four steps. The first step comprises oxidation of 2,2′-bipyridine **1** using 30% hydrogen peroxide in trifluoroacetic acid, in which 2,2′-bipyridine is oxidized to its corresponding *N*-oxide derivative **2**. In the second step the *N*-oxide intermediate is subjected to nucleophilic substitution with trimethylsilyl cyanide and benzoyl chloride to introduce the cyano group at the 6th position of the bipyridine ring, yielding (2,2′-bipyridine)-6-carbonitrile **3**. The third step is hydrolysis in which the cyano group is hydrolyzed using ethanol and sulfuric acid to yield ethyl (2,2′-bipyridine)-6-carboxylate **4**. Fourth step is the condensation reaction of ethyl (2,2′-bipyridine)-6-carboxylate with hydroxylamine hydrochloride in the presence of *N,N*′-diisopropylethylamine to yield *N*-hydroxy-(2,2′-bipyridine)-6-carboxamide **1A** (Scheme 1).

**Scheme 2:**
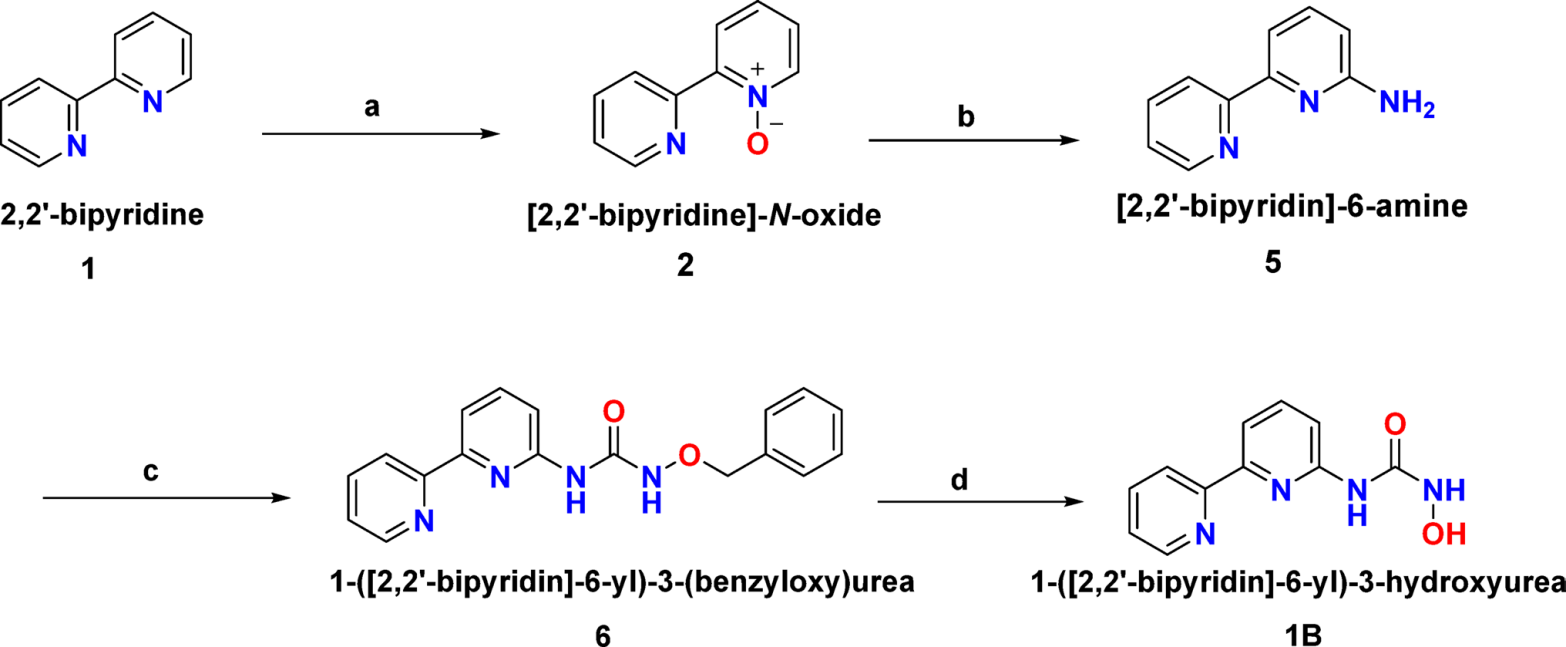
Chemical synthesis of compound **1B**, **a** trifluoroacetic acid, 30% hydrogen peroxide, room temperature, 2 h, **b** 2,2,2-trifluoro toluene, tertiary butyl amine, p-toluenesulfonyl chloride and trifluoroacetic acid, 90 °C, 24 h **c** *O*-benzyl hydroxylamine hydrochloride, 1,1′-carbonyldiimidazole, *N,N*′-diisopropylethylamine under N_2_ and **d** Palladium on carbon, ethanol, hydrogen, 60 psi, room temperature, 3 h

The synthesis of compound **1B** also involves four steps. The first step comprises the preparation of (2,2′-bipyridine)-*N*-oxide **2** by the oxidation of 2,2′-bipyridine **1**, using 30% hydrogen peroxide in trifluoroacetic acid. It was then reacted with tertiary butylamine and p-toluenesulfonyl chloride in 2,2,2-trifluoro toluene, followed by treatment with trifluoroacetic acid in the second step. This sequence of reactions leads to the formation of (2,2′-bipyridin)-6-amine **5**. Followed by a third step which involves a separate reaction in which *O*-benzyl hydroxylamine hydrochloride is reacted with 1,1′-carbonyldiimidazole and *N,N*' diisopropylethylamine to form an intermediate, which is then reacted with (2,2′-bipyridin)-6-amine. This step leads to the formation of 1-([2,2′-bipyridin]-6-yl)-3-(benzyloxy)urea **6**. Hydrogenation is the final step in which 1-([2,2′-bipyridin]-6-yl)-3-(benzyloxy)urea is subjected to hydrogenation using ethanol and palladium on carbon under hydrogen atmosphere (600 psi). This step is crucial for removing the benzyl protecting group and converting the benzyloxy group to a hydroxyl group, resulting in the formation of 1-(2,2′-bipyridin-6-yl)-3-hydroxyurea **1B** (Scheme 2).

The multi-step chemical syntheses of **1A** and **1B** involved the oxidation of 2,2′-bipyridine and subsequent reactions. Further, the structural characterization through NMR, FTIR, and HRMS confirms the successful synthesis of the derivatives, **1A** and **1B**.

### Pharmacology analysis, synthetic accessibility, and novelty of the developed compound prediction

The novelty of the compounds **1A** and **1B** was analysed by querying the chemical ligand structures on Pubchem and SciFinder, for the identification of similar structures. The search revealed that both **1A** and **1B**, were not present in the SciFinder database. An evaluation of synthetic accessibility was conducted to determine the likelihood and practicality of synthesizing the compounds. This assessment relied on screening and analysing information regarding the fragments’ contribution and the complexity of structures, sourced from a vast database of previously synthesized compounds. This predictive method allowed for the estimation of the synthetic accessibility of potential drug lead compounds through a de novo synthesis approach. It was found that **1A** and **1B** had synthetic accessibility values of 2.31 and 2.36 respectively. These values have almost similar synthetic accessibility values with respect to standard compound SAHA with the values 2.06 and 1.91 respectively. Subsequent in silico pharmacological analysis was conducted to assess the bioactivity of the compounds (**1A** and **1B**). Both the compounds adheres to Lipinski's rule of five, demonstrating drug-like characteristics (Tables 2 and 3).

**Table 2:** In silico pre-clinical trial using pkCSM and SWISS ADME

Parameters	1A	1B	SAHA
GI absorption (%)	71.58	92.49	87.11
BBB permeant (logBB)	− 0.50	− 1.23	− 0.74
Maximum tolerated dose (human) (log mg/kg/day)	0.50	0.55	0.001
Skin sensation	No	No	No
Fraction unbound (human) (Fu)	0.35	0.36	0.17
P-gP substrate	No	No	Yes
Renal OCT2 substrate	No	No	No
Oral rat acute toxicity (LD_50_) (mol/kg)	2.71	2.66	1.72
Oral rat chronic toxicity (log mg/kg_bw/day)	0.89	0.51	1.98

**Table 3:** In silico pharmacological properties

Physicochemical properties	1A	1B	SAHA
Chemical formula	C_11_H_9_N_3_O_2_	C_11_H_10_N_4_O_2_	C_14_H_20_N_2_O_3_
Molecular weight (g/mol)	215.21	230.22	264.32
Number of H-bond acceptors	4	4	3
Number of H-bond donors	2	3	3
Octanol–water partition coefficient (LogP)	1.90	0.92	1.84
Topological polar surface area (TPSA) (Å^2^)	75.11	87.14	78.43
Number of rotatable bonds	3	4	10
Violation of Lipinski rule of five	No	No	No

These *in-silico* analyses strengthen the present study by confirming the novelty of **1A** and **1B** emphasizing their potential as lead for anticancer drug design. Further, their adherence to Lipinski's rule of 5 and favourable pharmacological properties enhance their drug-like characteristics, setting the stage for future preclinical and clinical investigations.

### Biological evaluation

#### In-vitro cell viability assay

The cytotoxic effects of **1A and 1B** on three cancer cell lines, SiHa, Cal27, and MCF7 along with normal fibroblast cells was investigated. Parent moieties, 2,2′-Bipyridine and HU served as controls and SAHA served as a positive control in the assay. The summarized results, expressed as IC_50_ values, are presented in Table 4. The results demonstrated that **1A** and **1B** were less effective in SiHa cells with IC_50_ values exceeding 100 mΜ, compared to other two cancer cell lines MCF7 and Cal27. The compounds **1A** and **1B** were more potent in inducing cytotoxicity in Cal27 cell lines at an IC_50_ value of 19.36 and 35.31 μM respectively.

**Table 4:** IC_50_ values of tested compounds

Compound	Anti-proliferative activity IC_50_ (µM)			
	SiHa	MCF7	Cal27	Fibroblast
**1A**	> 100	74.14 ± 1.10	19.36 ± 0.91	> 100
**1B**	> 100	54.21 ± 3.53	35.31 ± 3.16	> 100
2,2′-Bipyridine	> 100	> 100	> 100	> 100
HU	> 100	> 100	> 100	> 100
SAHA	25.16 ± 3.69	> 100	10.80 ± 4.80	> 100

#### Wound healing assay

**Fig. 3:**
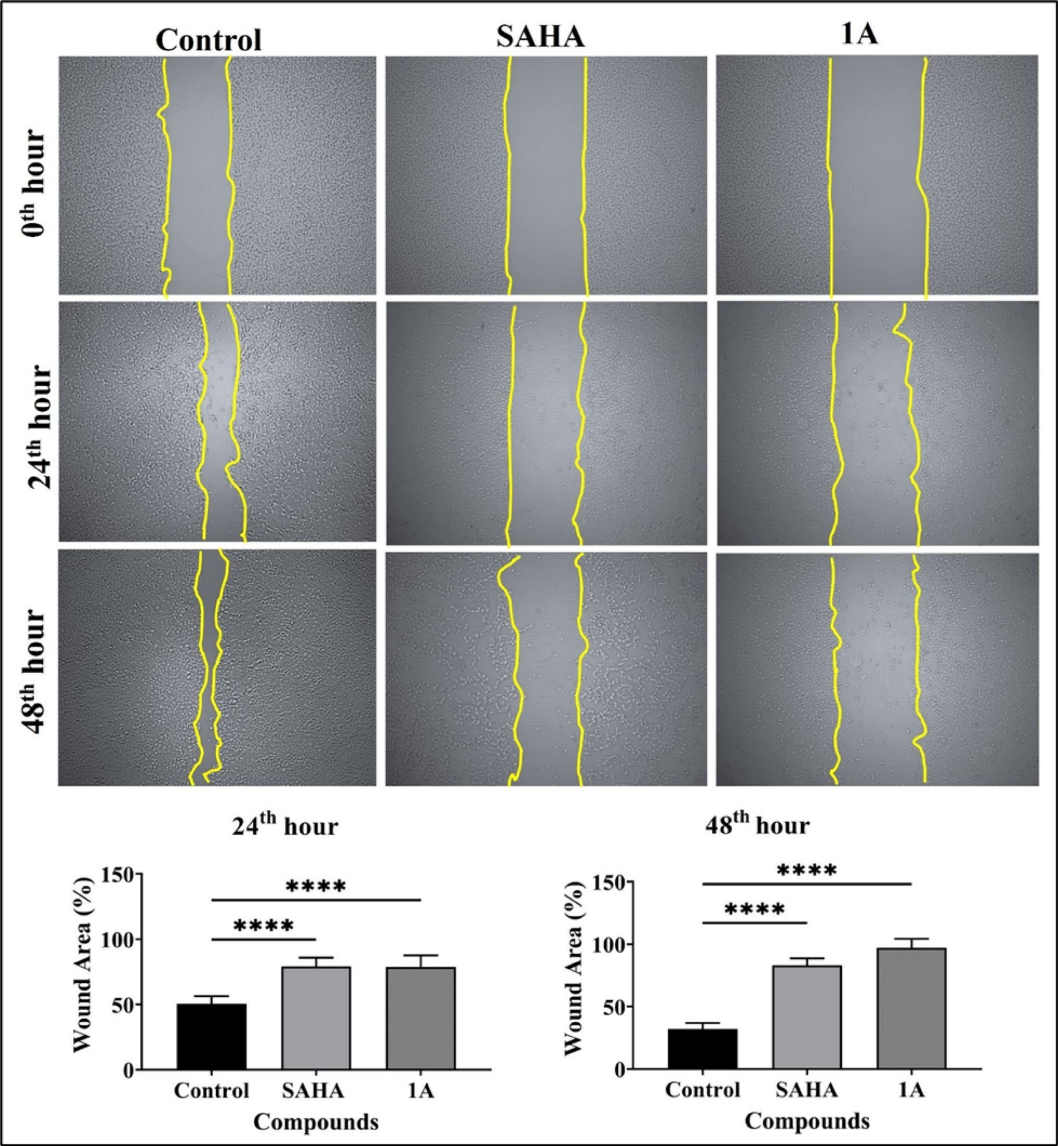
Wound healing assay in Cal27 cells following treatment with SAHA and **1A** at their respective IC_50_ concentrations for 24 h. Images of cell monolayers wounded by tip (200 µL) in untreated control and treatment groups; Quantitative analysis of percentage of wound area is represented as bar graph. Statistical differences were analyzed with a one-way ANOVA post-hoc Dunnet’s test. ****p < 0.0001 compared with untreated control. Data represented are the mean ± SD from an independent set of experiments

Majority of the cancers are difficult to treat due to their faster migration and invasion through the extracellular matrix into the systemic circulation which enables them to travel and attach themselves to distant locations and proliferate. To investigate the ability of the Cal27 cells to undergo metastatic progression an in vitro wound healing assay was performed upon treatment of Cal27 cells with **1A** and positive control SAHA at their respective IC_50_ concentrations at 24 and 48 h compared to the untreated control cells. Cal27 cells treated with compound **1A** exhibited slightly better antimigratory effect in comparison to positive control SAHA at 48th hour. The data suggests that compound **1A** inhibits cell motility and thereby cell migration in Cal27 cells (Fig. 3).

#### Cell cycle analysis

**Fig. 4:**
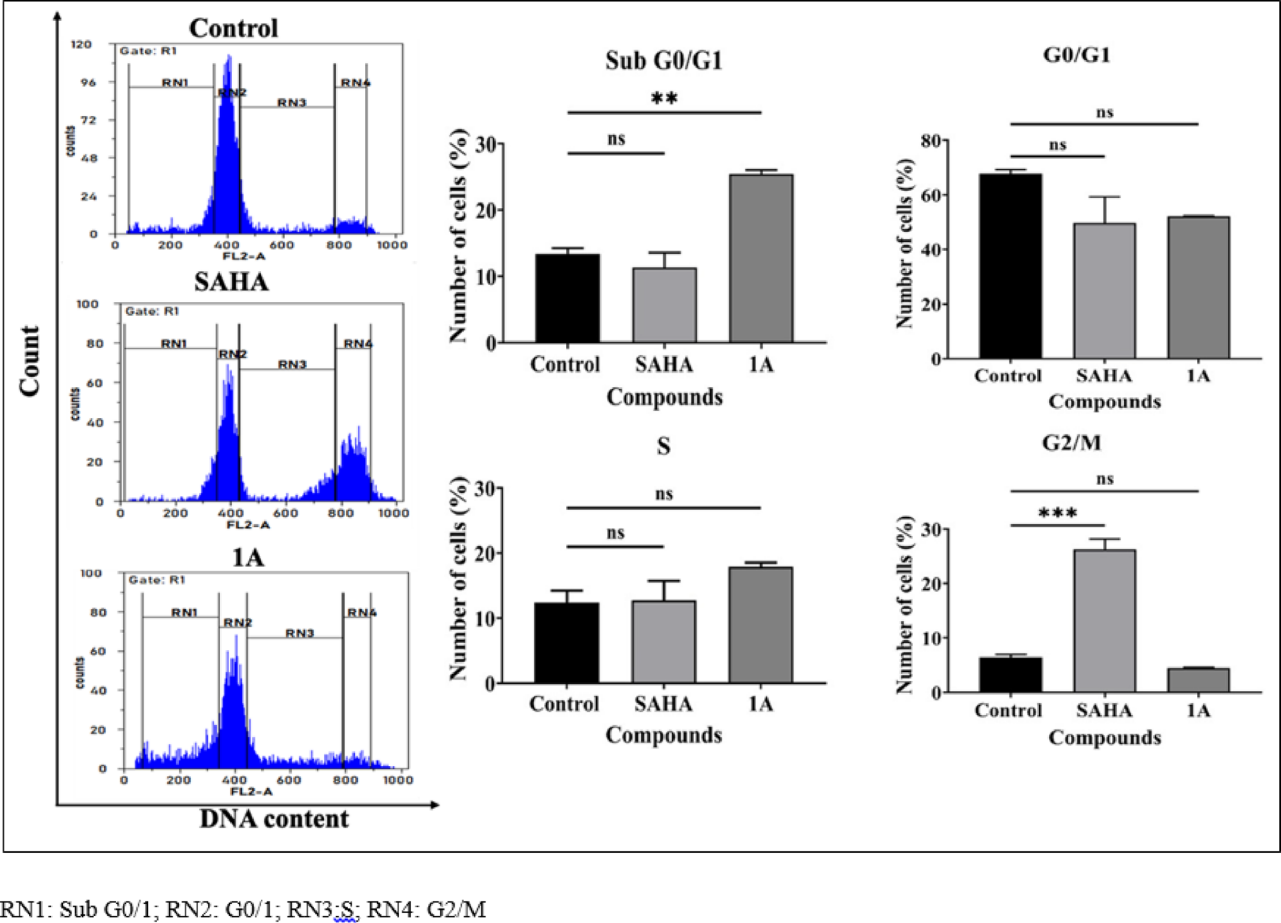
Cell cycle studies for Cal27 cells following treatment with SAHA and **1A** at their respective IC_50_ concentrations for 48 h by flow cytometry. Representative histograms after deoxyribonucleic acid (DNA) staining are presented on the left panel; Quantification of cell cycle analysis and statistical analyses of histograms are presented on the right panel. Statistical differences were analysed with a one-way ANOVA post-hoc Dunnet’s test. **p < 0.01; ***p < 0.001; ns, non-significant compared with untreated control. Data represented are the mean ± SD from an independent set of experiments

The anticancer efficacy of the compounds is intricately linked to the cell cycle. The impact of **1A** and positive control SAHA at their respective IC_50_ concentrations for 48 h on the cell cycle distribution in Cal27 cells using flow cytometry was investigated. The findings revealed that compound **1A** caused a notable increase in the percentage of Cal27 cells in the sub G0/G1 phase, rising from 13.39% to 25.43%, compared to the control (Fig. 4).

#### Apoptosis

**Fig. 5:**
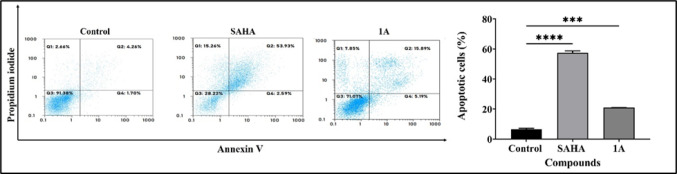
Apoptosis studies for Cal27 cells following treatment with SAHA and **1A** at their respective IC_50_ concentrations for 48 h by flow cytometry. Representative scatter plots are presented on the left panel; Quantification of apoptosis and statistical analyses of scatter plots are presented on the right panel. Statistical differences were analysed with a one-way ANOVA post-hoc Dunnet’s test. ***p < 0.001; ****p < 0.0001 compared with untreated control. Data represented are the mean ± SD from an independent set of experiments

The cell cycle analysis revealed an increase in the cell count in the sub-G0/1 phase. To further investigate whether cell death is a result of physiological apoptosis or non-specific necrosis, Annexin V/PI dual staining was conducted. Cal27 cells were treated with positive control SAHA and **1A** at their respective IC_50_ concentrations for 48 h, and the analysis focused on their ability to induce early apoptosis, late apoptosis, and necrosis. The apoptotic analysis demonstrated that the percentage of cell apoptosis increased from 6.47% in untreated Cal27 cells to 21.08% in cells treated with compound **1A**. The results indicated a higher proportion of late apoptosis compared to early apoptosis, suggesting that compound **1A** induces irreversible apoptosis (Fig. 5).

#### Reactive oxygen species (ROS) detection

**Fig. 6:**
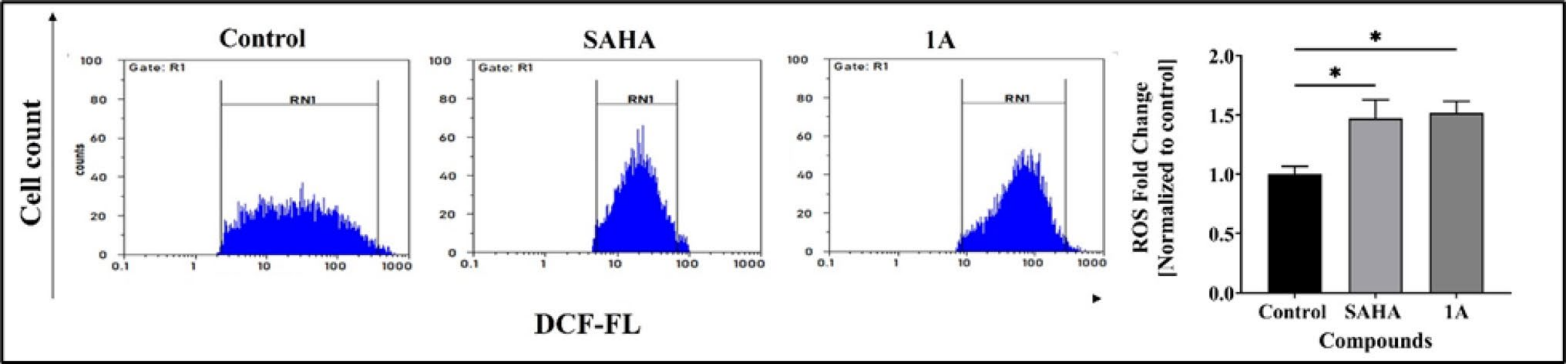
ROS studies for Cal27 cells following treatment with SAHA and **1A** at their respective IC_50_ concentrations for 48 h by flow cytometry. Representative histograms are presented on the left panel; Quantification of ROS accumulation and statistical analyses of histograms are presented on the right panel. Statistical differences were analysed with a one-way ANOVA post-hoc Dunnet’s test. *p < 0.05 compared with untreated control. Data represented are the mean ± SD from an independent set of experiments

ROS play a crucial role in the regulation and induction of cellular apoptosis in cancer cells. Therefore, the mechanism of apoptotic induction was explored by the analysis of intracellular ROS levels upon treatment with positive control SAHA and **1A** at their IC_50_ concentrations in the Cal27 cells respectively. The cells were stained using DCFH-DA. Treatment of Cal27 cells with compound **1A** resulted in a significant increase in ROS accumulation, with a fold change of 1.52 as compared to the untreated control (Fig. 6).

#### Colony formation assay

**Fig. 7:**
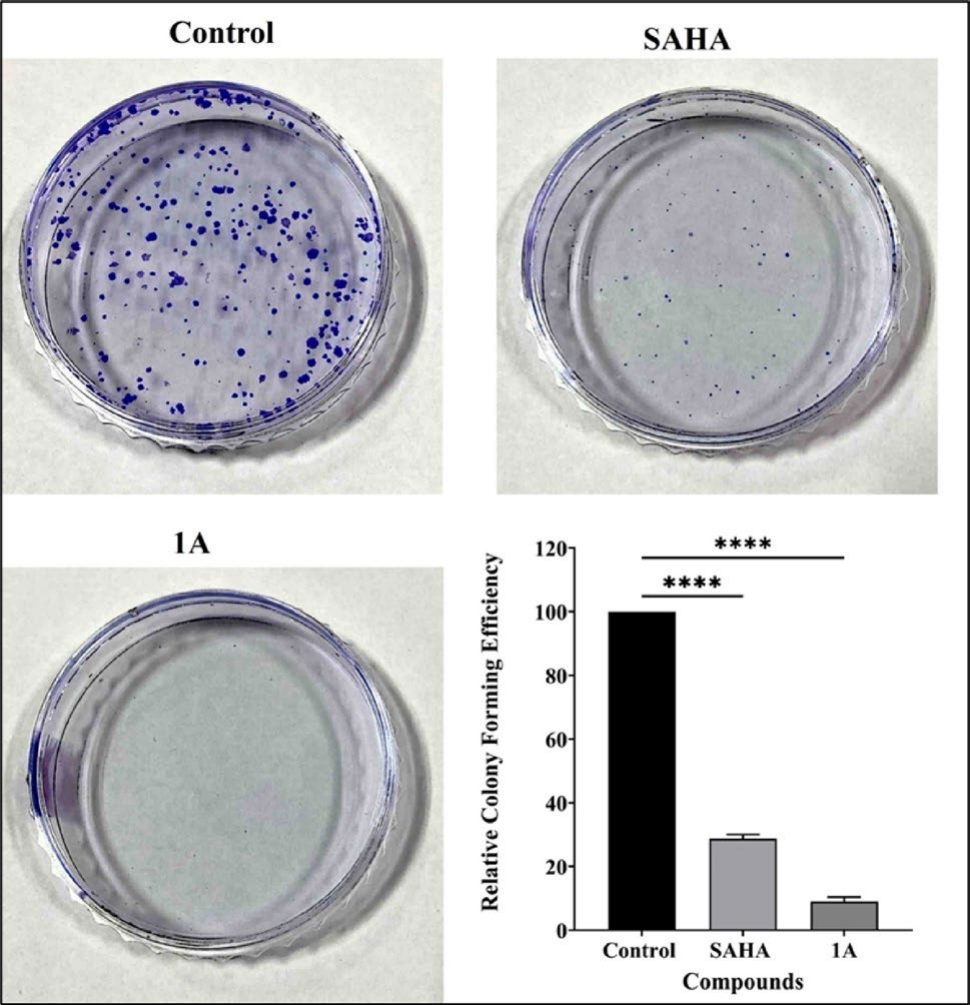
Colony formation assay in Cal27 cells following treatment with SAHA and **1A** at their respective IC_50_ concentrations for 48 h. Images of Cal27 cells stained with crystal violet; Quantitative analysis of cell colony numbers are represented as bar graph. Statistical differences were analysed with a one-way ANOVA post-hoc Dunnet’s test. ****p < 0.0001 compared with untreated control. Data represented are the mean ± SD from an independent set of experiments

The colony formation assay is used to assess cell survival by evaluating the ability of individual cells to grow into colonies. This assay is crucial for determining the tumorigenic potential of cancer cells in vitro. The assay results demonstrated that the number of colonies significantly decreased in **1A** treated Cal27 cells, better than positive control SAHA. This indicates that compound **1A** reduced the proliferation of Cal27 cells (Fig. 7).

#### HDAC inhibitory assay

HDAC inhibitory activity of compound **1A** was performed by an in vitro fluorescence based assay using SAHA as positive control. Analysis of the obtained results revealed that **1A** showed HDAC inhibitory activity with the IC_50_ value of 28.97 µM. However, the positive control SAHA displayed the potent HDAC inhibition with the IC_50_ value of 0.17 µM (Table 5).

**Table 5:** Inhibitory effects of **1A** and SAHA on HDAC activity

Compound code	HDAC activity (IC_50_ µM)
**1A**	28.97
SAHA	0.17

The biological assessment of the two compounds revealed that compound **1A** is comparatively better compound, exhibiting potent anti-proliferative activity against an oral cancer Cal27 cells better than the efficacy of the parent moieties HU and 2,2′-Bipyridine. Thus, compound **1A** emerges as an effective anticancer agent. The wound healing assay further substantiated its anti-cancer properties, demonstrating a significant inhibitory effect on cancer cell migration. The induction of cell death, elucidated through cell cycle analysis and apoptosis studies, distinguished **1A** by triggering both early and late apoptosis, a crucial characteristic for targeted cancer therapy. The study investigated the mechanisms underlying **1A** induced apoptosis, highlighting the key role of ROS. The significant increase in ROS generation in Cal27 cells following **1A** treatment suggested a potential association between apoptosis and oxidative stress. Furthermore, the incorporation of HDAC inhibitory activity assessment supports with the contemporary interest in epigenetic modulation for cancer treatment. **1A**’s effective inhibition of HDAC activity, evident in the in vitro assay, places it as a promising candidate in the scope of epigenetic therapies. Further, the molecular docking studies and subsequent MD simulations provide dynamic insights into the interaction between **1A** and different HDAC isoforms.

### Molecular docking studies

**Fig. 8:**
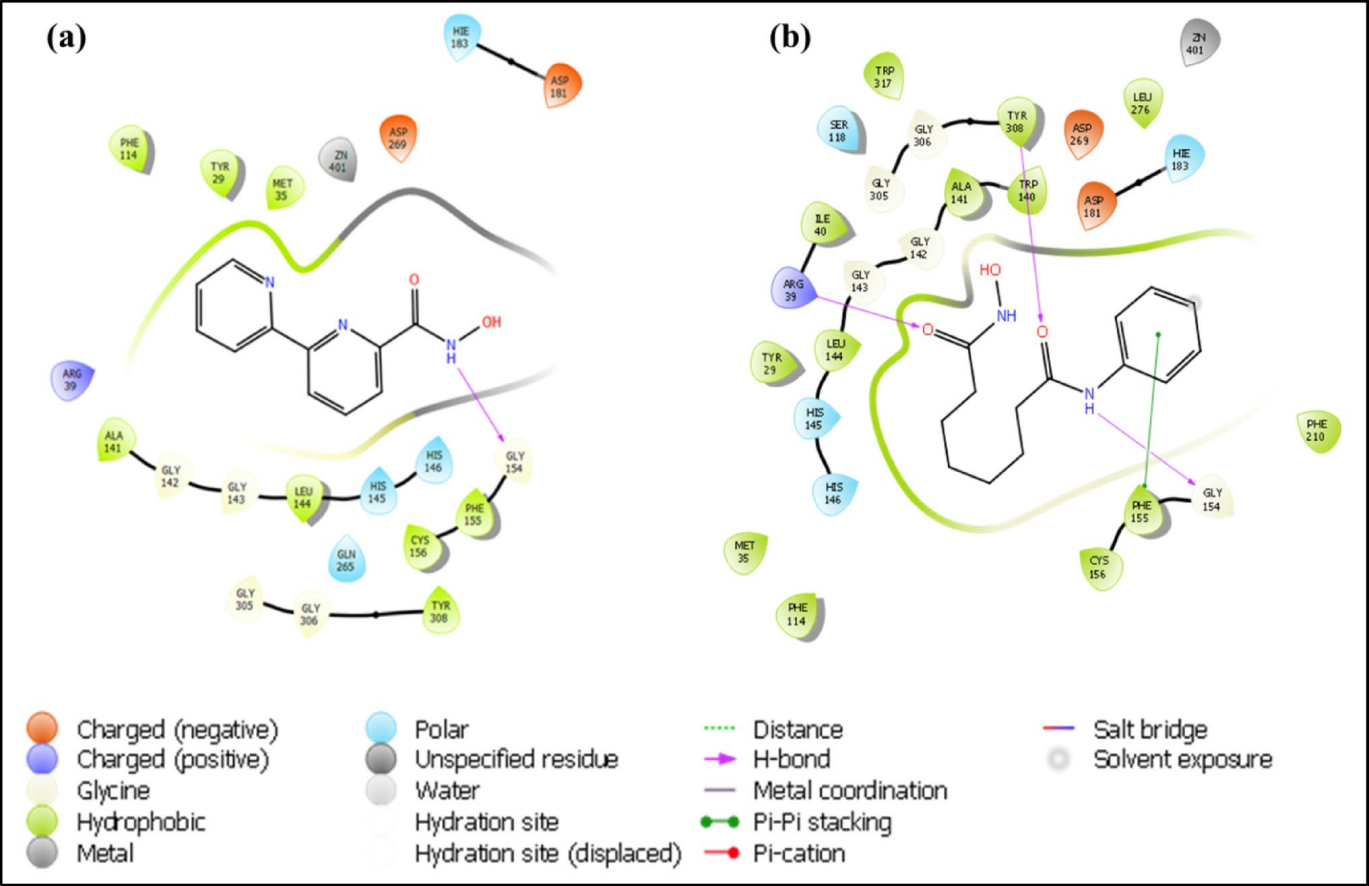
Binding mode of studied compounds retrieved from focused molecular docking predicting the 2D binding mode and receptor–ligand interaction diagrams of HDAC 2 (PDB ID: 4LY1) **a** with **1A** and **b** SAHA

In silico molecular docking studies were also performed to support the in vitro HDAC inhibitory results and to understand the inhibitory activity of **1A** against different HDAC isoforms using a validated molecular docking program (Maestro version 11.4 Schrodinger Inc.). Compounds were docked into the active sites of HDAC1-11. Known Food and Drug Administration (FDA) approved hydroxamic acid-based HDAC inhibitor SAHA was also subjected to docking studies. The results showed that **1A** could interact with all the isoforms of classical HDACs (Table 6). However, **1A** showed a maximum binding affinity towards HDAC 2 with a free binding energy of − 10.28 kcal/mol, followed by HDAC 8 (− 8.59 kcal/mol). It is comparable with the maximum binding energy of SAHA towards HDAC 2 with the docking score of − 12.04 kcal/mol. Having a comparatively better interaction with HDAC 2, the 2D interaction data of **1A** and positive control SAHA with HDAC 2 protein is disclosed and both show that their hydroxamate group forms a metal ion interaction with the catalytic Zn^2+^ ion (Fig. 8 and Table 7).

**Table 6:** XP dock scores of the compounds with all the classical HDAC isoforms and energy (Δ) in kcal/mol

Compound	*Isoform*	HDAC 1	HDAC 2	HDAC 3	HDAC 4	HDAC 5	HDAC 6	HDAC 7	HDAC 8	HDAC 9	HDAC 10	HDAC 11
	*PDB ID*	(4BKX)	(4LY1)	(4A69)	(2VJQ)	(Q9UQL6)	(3PHD)	(3ZNR)	(1T69)	(Q9UKV0)	(6UII)	(Q96DB2)
1A		− 4.08	− 10.28	− 2.24	− 6.50	− 3.13	− 5.57	− 7.70	− 8.59	− 3.95	− 5.66	− 6.12
SAHA		− 4.43	− 12.04	− 1.18	− 8.29	− 1.17	− 3.52	− 8.24	− 9.91	− 2.24	− 8.97	− 7.73

**Table 7:** Ligand (**1A** and SAHA) interactions with HDAC 2

Ligand	Hydrophobic interaction	H-bond interaction	Polar interaction
1A	TYR 29, MET 35, PHE 114, ALA 141, LEU 144, PHE 155, CYS 156, TYR 308	GLY 154	HIS 145, HIS 146, HIE 183, GLN 265
SAHA	TYR 29, MET 35, ILE 40, PHE 114, TRP 140, ALA 141, LEU 144, PHE 155, CYS 156, PHE 210, LEU 276, TYR 308, TRP 317	ARG 39, GLY 154, TYR 308	SER 118, HIS 145, HIS 146, HIE 183

### Molecular dynamic simulation

**Fig. 9:**
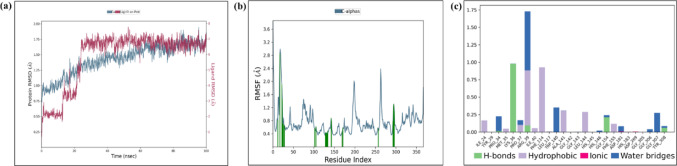
**a** Plot presenting the stability of protein–ligand interaction (RMSD), **b** The protein conformation changes along its side chain are represented in the RMSF throughout the trajectory and **c** Protein–ligand contact histogram of **1A** complexed with HDAC 2 during 100 ns molecular dynamics simulation

Molecular docking studies are limited to predicting ligand interactions at the receptor active site in static conditions, providing docking poses without capturing dynamic atomic movements over time. To address this, MD simulations utilize Newton’s equation of motion to estimate atomic motions over time, providing insights into the ligand’s binding state under physiological conditions. Following the determination of the biological activities of compound **1A** and molecular docking results, **1A**, exhibiting the best XP dock score (− 10.28) toward HDAC 2, was subjected to 100 ns MD simulation to predict its affinity for HDAC 2. MD trajectory analysis was employed to assess the interaction between protein and ligand interactions, deviation from the root mean square value (RMSD), and root mean square fluctuations (RMSF). RMSD and RMSF provide insights into the equilibrium and fluctuations of the protein complex throughout the simulation. The RMSD plot for **1A**-HDAC 2 (Fig. 9a) revealed slight drifts during specific time intervals (20 ns, 25–40 ns, and 40–60 ns) but overall stability from 60 to 100 ns during the simulation was observed. Conversely, Fig. 9b illustrated conformational changes in the HDAC 2 protein side chain throughout the simulation, with RMSF data showing flexibility ranging from 0.4 to 2.4 Å. Figure 9c depicts the overall interaction between the HDAC 2 protein and **1A** during the simulation.

The stable binding of **1A** to HDAC 2 with a maximum binding affinity, highlights the potential of compound **1A** as a promising selective HDAC inhibitor and offers a molecular basis for its observed HDAC inhibitory activities.

## Conclusion

This study successfully designed and synthesized two novel 2,2′-bipyridine hydroxamic acid derivatives (**1A** and **1B**) as potential HDAC targeting anticancer agents for HNC. Through comprehensive biological evaluation, compound **1A** demonstrated better selective cytotoxicity against oral cancer Cal27 cells (IC_50_ = 19.36 μM) compared to parental moieties hydroxyurea and 2,2′-bipyridine, while showing minimal toxicity to normal fibroblasts. Further biological assessments revealed that compound **1A** effectively inhibited cancer cell migration in wound healing assays, induced substantial ROS-mediated apoptosis (21.08% vs. 6.47% in controls), and caused significant cell cycle arrest in the sub-G0/G1 phase (25.43% *vs* 13.39% in controls). Particularly, **1A** exhibited moderate but selective HDAC inhibitory activity (IC_50_ = 28.97 μM), with molecular docking and 100 ns MD simulations confirming stable binding to HDAC 2’s catalytic domain through crucial Zn^2+^ co-ordination by its hydroxamic acid moiety, similar to SAHA but with distinct interaction patterns. In addition, previously reported HU derivatives such as BOU, MHCU, and Mito-HUs have shown broad-spectrum antiproliferative effects. While compound **1A** demonstrates minimal toxicity to normal cells, and selective HDAC inhibition suggesting a more refined and specific anticancer profile. Furthermore, compound’s favourable drug-like properties, including good GI absorption (71.58%) and synthetic accessibility (score 2.31), along with its epigenetic (HDAC inhibition) and cytotoxic (ROS induction) mechanisms, place **1A** as a promising lead compound worthy of further development. However, future studies should explore structure–activity relationships through analogue synthesis, in vivo efficacy in HNC xenograft models, and potential combination therapies with existing chemotherapeutic agents to enhance its clinical translation potential for treating HNC and other malignancies characterized by HDAC overexpression.

## Electronic supplementary material

Below is the link to the electronic supplementary material.

Supplementary Material 1

## References

[1] Gormley M, Creaney G, Schache A, Ingarfield K, Conway DI (2008) Reviewing the epidemiology of head and neck cancer: definitions, trends and risk factors. Br Dent J. 10.1038/s41415-022-5166-x 10.1038/s41415-022-5166-x PMC9652141 36369568

[2] Bugshan A, Farooq I (2020) Oral squamous cell carcinoma: metastasis, potentially associated malignant disorders, etiology and recent advancements in diagnosis. F100Research. 10.12688/f1000research.22941.1 10.12688/f1000research.22941.1 PMC7194458 32399208

[3] Markopoulos AK (2012) Current aspects on oral squamous cell carcinoma. Open Dent J. 10.2174/1874210601206010126 22930665 10.2174/1874210601206010126 PMC3428647

[4] Chinn SB, Myers JN (2015) Oral cavity carcinoma: current management, controversies, and future directions. J Clin Oncol. 10.1200/JCO.2015.61.2929 26351335 10.1200/JCO.2015.61.2929 PMC5320919

[5] National Cancer Institute (2024) Drugs Approved for Head and Neck Cancer, U.S. https://www.cancer.gov/about-cancer/treatment/drugs/head-neck#drugs-approved-for-head-and-neck-cancer. Accessed 4 June 2025

[6] Adhikari S, Nath P, Das A, Datta A, Baildya N, Duttaroy AK, Pathak S (2024) A review on metal complexes and its anticancer activities: Recent updates from in vivo studies. Biomed Pharmacother. 10.1016/j.biopha.2024.116211 38290253 10.1016/j.biopha.2024.116211

[7] Jinna S, Khandhar PB (2019) Hydroxyurea toxicity. StatPearls Publishing, Treasure Island 30725894

[8] Perković I, Butula I, Zorc B, Hock K, Pavelić SK, Pavelić K, Clercq ED, Balzarini J, Mintas M (2008) Novel lipophilic hydroxyurea derivatives: synthesis, cytostatic and antiviral activity evaluations. Chem Biol Drug. 10.1111/j.1747-0285.2008.00660.x 10.1111/j.1747-0285.2008.00660.x 18410308

[9] Šaban N, Stepanić V, Vučinić S, Horvatić A, Cindrić M, Perković I, Zorc B, Oršolić N, Mintas M, Pavelić K, Pavelić SK (2013) Antitumor mechanisms of amino acid hydroxyurea derivatives in the metastatic colon cancer model. Int J Mol Sci. 10.3390/ijms141223654 24304540 10.3390/ijms141223654 PMC3876069

[10] Cheng G, Hardy M, Topchyan P, Zander R, Volberding P, Cui W, Kalyanaraman B (2021) Mitochondria-targeted hydroxyurea inhibits OXPHOS and induces antiproliferative and immunomodulatory effects. iScience. 10.1016/j.isci.2021.102673 34189437 10.1016/j.isci.2021.102673 PMC8215227

[11] Opačić N, Barbarić M, Zorc B, Cetina M, Nagl A, Frković D, Kralj M, Pavelić K, Balzarini J, Andrei G, Snoeck R (2005) The novel L-and D-amino acid derivatives of hydroxyurea and hydantoins: synthesis, X-ray crystal structure study, and cytostatic and antiviral activity evaluations. J Med Chem. 10.1021/jm040869i 15658861 10.1021/jm040869i

[12] Xu M, Hou Y, Li N, Yu W, Chen L (2024) Targeting histone deacetylases in head and neck squamous cell carcinoma: molecular mechanisms and therapeutic targets. J Transl Med. 10.1186/s12967-024-05169-9 38702756 10.1186/s12967-024-05169-9 PMC11067317

[13] Chemical Abstracts Service (CAS) (2024). Structure Search—SciFinderⁿ Training and Support, U.S. https://www.cas.org/support/training/scifinder-n/structure-search. Accessed 26 March 2024

[14] Daina A, Michielin O, Zoete V (2017) SwissADME: a free web tool to evaluate pharmacokinetics, drug-likeness and medicinal chemistry friendliness of small molecules. Sci Rep. 10.1038/srep42717 28256516 10.1038/srep42717 PMC5335600

[15] Vp DE, Blundell TL, Ascher DB (2015) pkCSM: predicting small-molecule pharmacokinetic properties using graph-based signatures. J Med Chem. 10.1021/acs.jmedchem.5b00104 10.1021/acs.jmedchem.5b00104 PMC4434528 25860834

[16] Mosmann T (1983) Rapid colorimetric assay for cellular growth and survival: application to proliferation and cytotoxicity assays. J Immunol Methods. 10.1016/0022-1759(83)90303-4 6606682 10.1016/0022-1759(83)90303-4

[17] Eldehna WM, Abo-Ashour MF, Ibrahim HS, Al-Ansary GH, Ghabbour HA, Elaasser MM, Ahmed HY, Safwat NA (2018) Novel [(3-indolylmethylene) hydrazono] indolin-2-ones as apoptotic anti-proliferative agents: design, synthesis and in vitro biological evaluation. J Enzyme Inhib Med Chem. 10.1080/14756366.2017.1421181 29560733 10.1080/14756366.2017.1421181 PMC6010103

[18] Almahli H, Hadchity E, Jaballah MY, Daher R, Ghabbour HA, Kabil MM, Al-Shakliah NS, Eldehna WM (2018) Development of novel synthesized phthalazinone-based PARP-1 inhibitors with apoptosis inducing mechanism in lung cancer. Bioorg Chem. 10.1016/j.bioorg.2018.01.034 29453076 10.1016/j.bioorg.2018.01.034

[19] Qian J, Xu Z, Meng C, Liu J, Hsu PL, Li Y, Zhu W, Yang Y, Morris-Natschke SL, Lee KH, Zhang Y (2020) Design and synthesis of benzylidenecyclohexenones as TrxR inhibitors displaying high anticancer activity and inducing ROS, apoptosis, and autophagy. Eur J Med Chem. 10.1016/j.ejmech.2020.112610 32736231 10.1016/j.ejmech.2020.112610

[20] He F, Ran Y, Li X, Wang D, Zhang Q, Lv J, Yu C, Qu Y, Zhang X, Xu A, Wei C (2020) Design, synthesis and biological evaluation of dual-function inhibitors targeting NMDAR and HDAC for Alzheimer’s disease. Bioorg Chem. 10.1016/j.bioorg.2020.104109 32768741 10.1016/j.bioorg.2020.104109

[21] Pai P, Kumar A, Shetty MG, Kini SG, Krishna MB, Satyamoorthy K, Babitha KS (2022) Identification of potent HDAC 2 inhibitors using E-pharmacophore modelling, structure-based virtual screening and molecular dynamic simulation. J Mol Model. 10.1007/s00894-022-05103-0 35419753 10.1007/s00894-022-05103-0 PMC9007783

