# Supplementary material for: Anticancer potential of 2,2′-bipyridine hydroxamic acid derivatives in head and neck cancer therapy
Source: J Comput Aided Mol Des. 2025 Aug 6;39(1):62. doi: 10.1007/s10822-025-00640-1 (PMC12328545; doi:10.1007/s10822-025-00640-1)
Supplement: Supplementary file 1 — Supplementary Material 1 [file 10822_2025_640_MOESM1_ESM.docx]

**Supporting Information**

**Anticancer Potential of 2,2**'**-Bipyridine Hydroxamic Acid Derivatives in Head and Neck Cancer Therapy**

Manasa Gangadhar Shetty^1^, Bipasa Dey^1^, Padmini Pai^1^, Babitha Kampa Sundara^1^*, Kapaettu Satyamoorthy^2^, Srinivas Oruganti^3^, Usha Yogendra Nayak^4^, Ashwini T^4^

^1^Department of Biophysics, Manipal School of Life Sciences, Manipal Academy of Higher Education, Manipal 576104, Karnataka, India

^2^Shri Dharmasthala Manjunatheshwara (SDM) University, Manjushree Nagar, Sattur, Dharwad, 580009, Karnataka, India.

^3^Dr. Reddy’s Institute of Life Sciences, University of Hyderabad Campus, Gachibowli 500046, Hyderabad, India.

^4^Department of Pharmaceutics, Manipal College of Pharmaceutical Sciences, Manipal Academy of Higher Education, Manipal 576104, Karnataka, India

*Corresponding author

Dr. Babitha K S

Associate Professor and Head

Department of Biophysics

Manipal School of Life Sciences

Manipal Academy of Higher Education

Manipal 576104, Karnataka, India

E-mail address: babitha.ks@manipal.edu

ORCID: <https://orcid.org/0000-0002-9385-9353>

**Chemical Structures of novel hydroxamic acids (1A and 1B)**

**
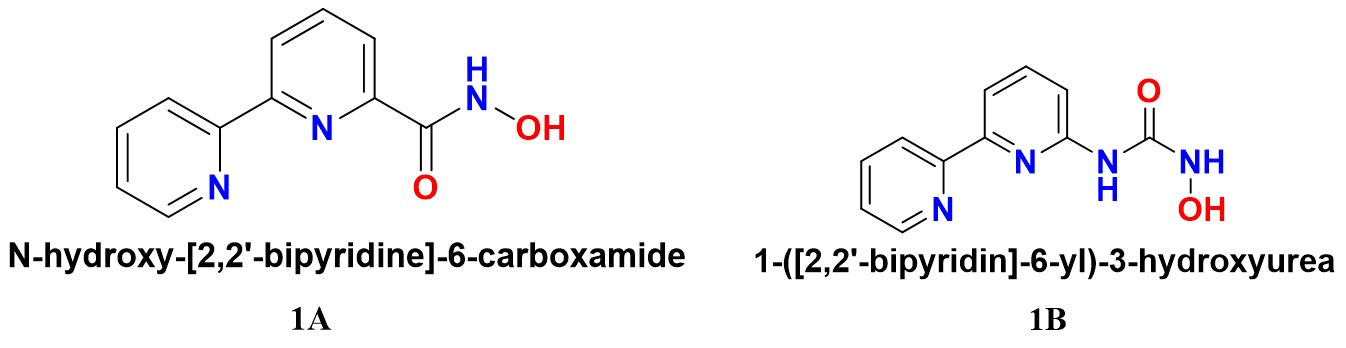
**

**Scheme 1:**


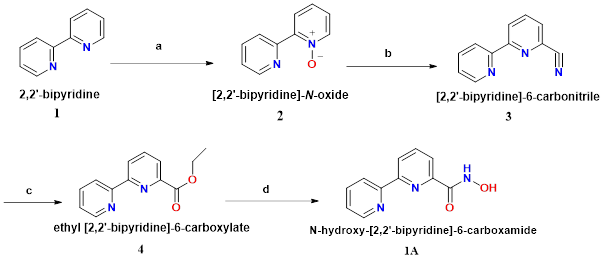


| **^1^H NMR spectrum of compound 2** |
| --- |
| 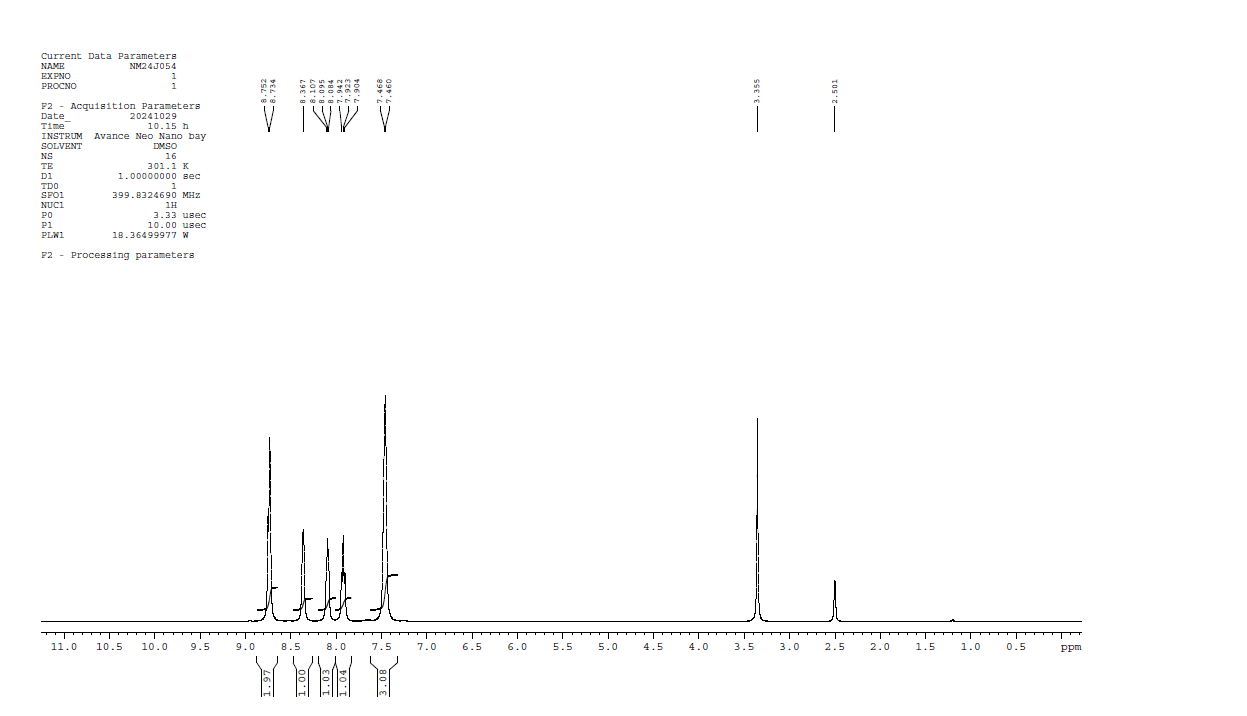 |
| **^13^C NMR spectrum of compound 2** |
| 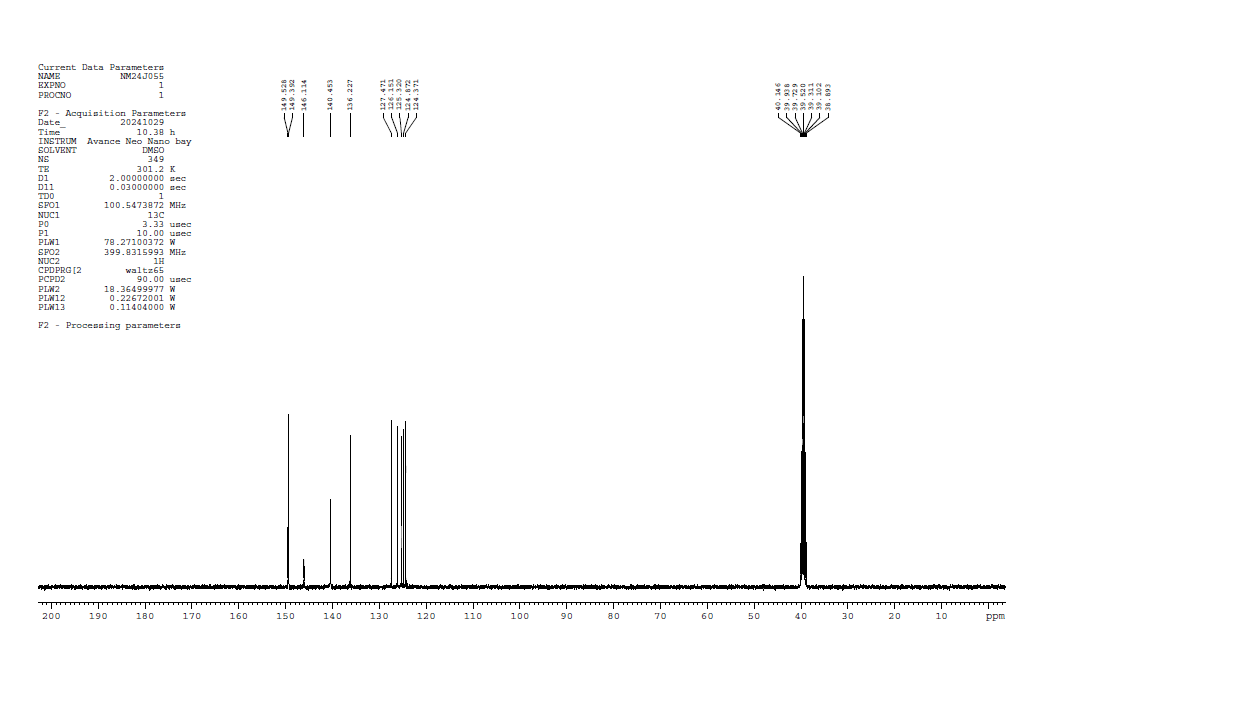 |
| **Mass spectrum of compound 2** |
|  |
| **HPLC chromatogram of compound 2** |
|  |
| **^1^H NMR spectrum of compound 3** |
| 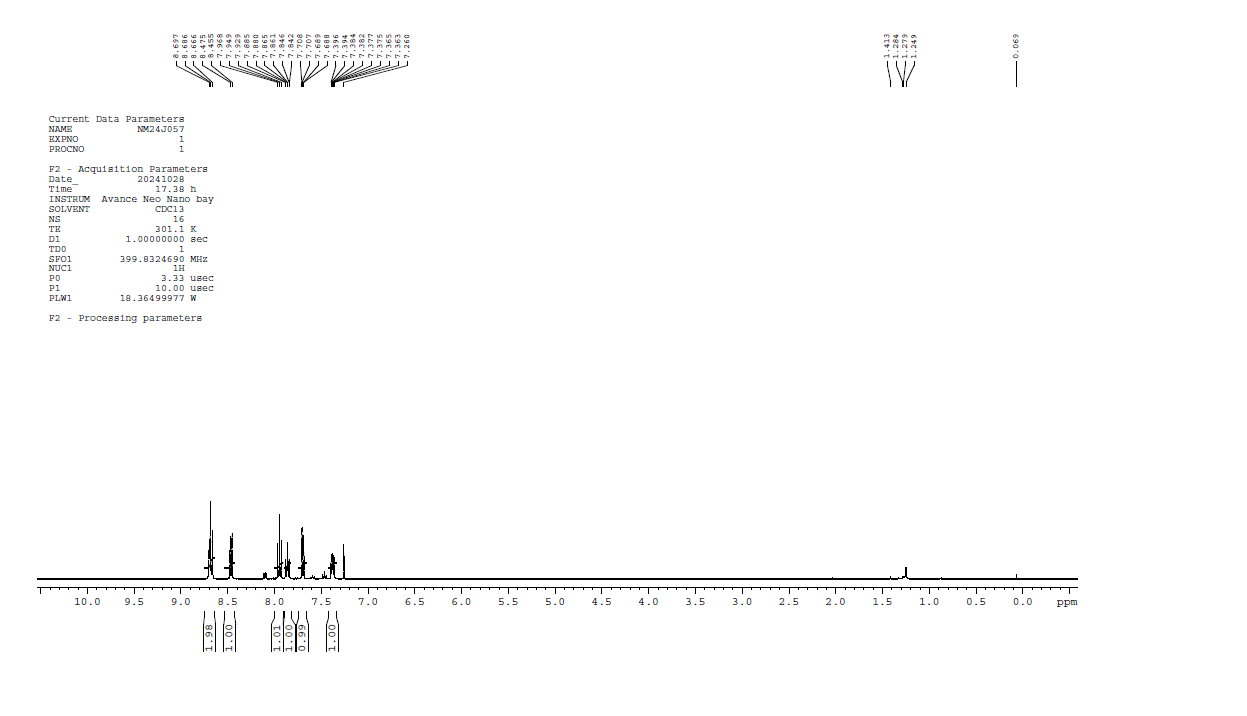 |
| **^13^C NMR spectrum of compound 3** |
| **^^** |
| **Mass spectrum of compound 3** |
| **^^** |
| **HPLC chromatogram of compound 3** |
| 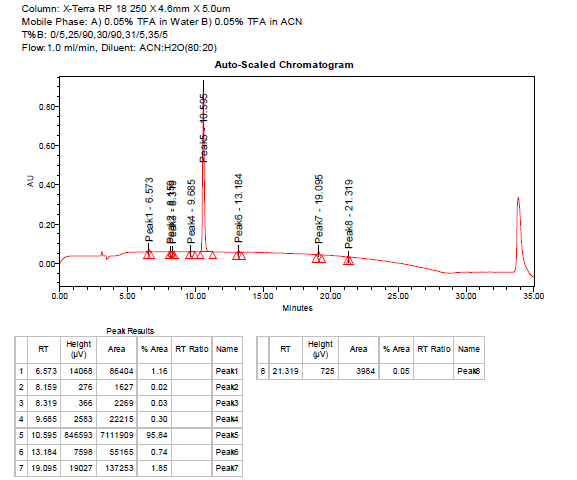 |
| **^1^H NMR spectrum of compound 4** |
| 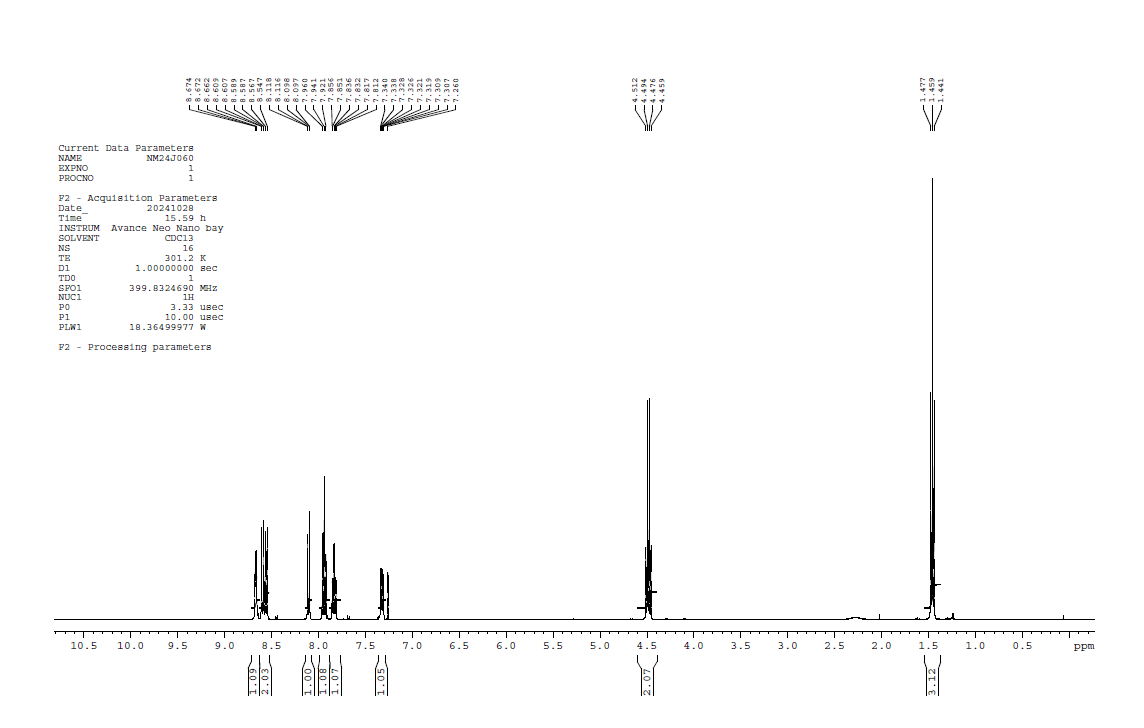 |
| **^13^C NMR spectrum of compound 4** |
| 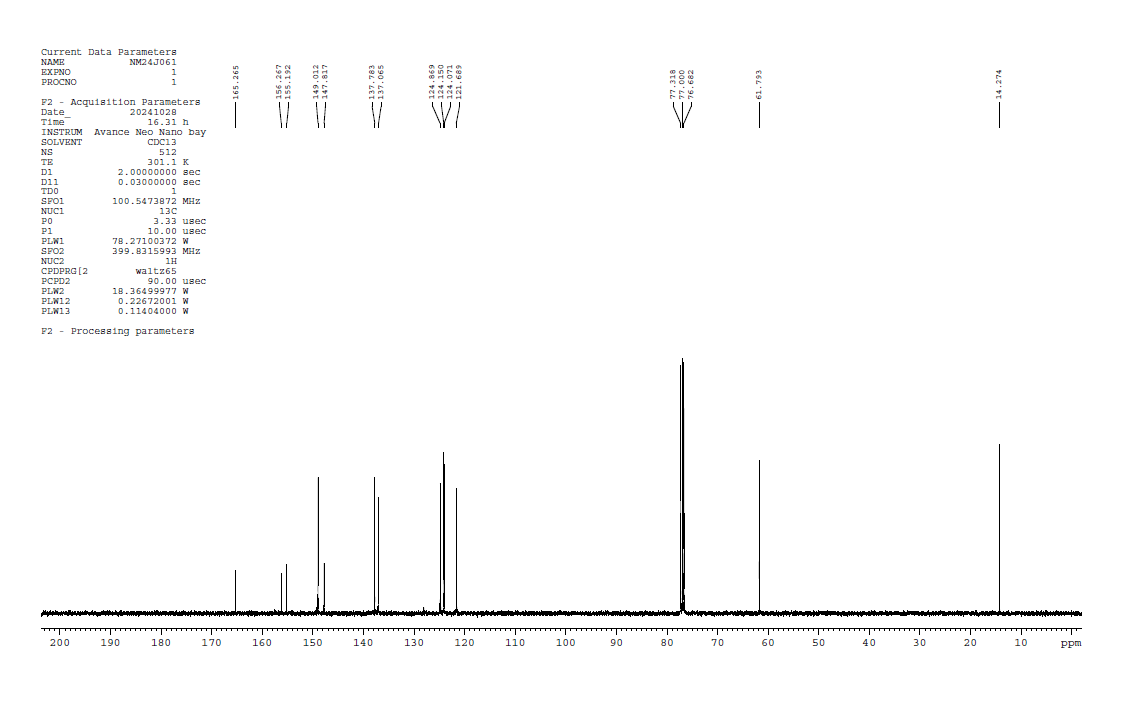 |
| **Mass spectrum of compound 4** |
| **^^** |
| **HPLC chromatogram of compound 4**  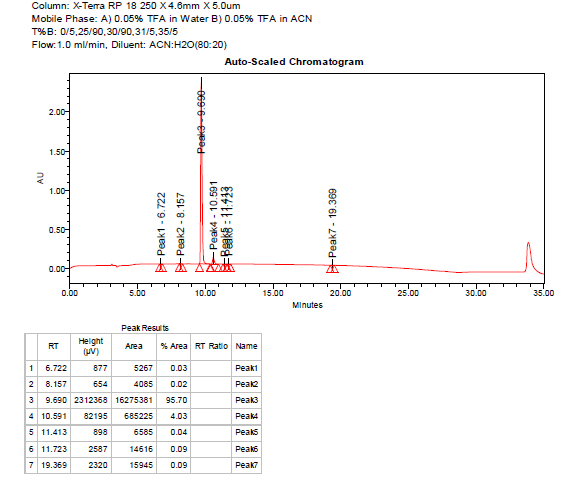 |
| **^1^H NMR spectrum of compound 1A** |
| **^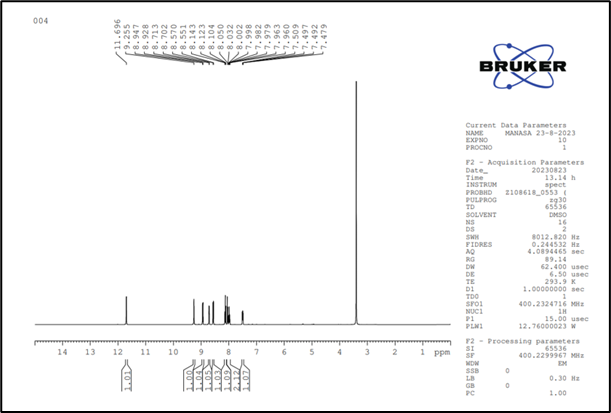^** |
| **^13^C NMR spectrum of compound 1A** |
| **^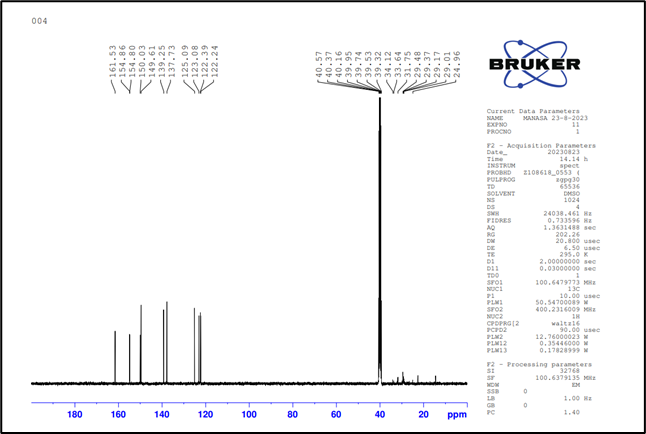^** |
| **Mass spectrum of compound 1A** |
| **^^** |
| **HPLC chromatogram of compound 1A** |
| **** |
| **FTIR of compound 1A** |
| 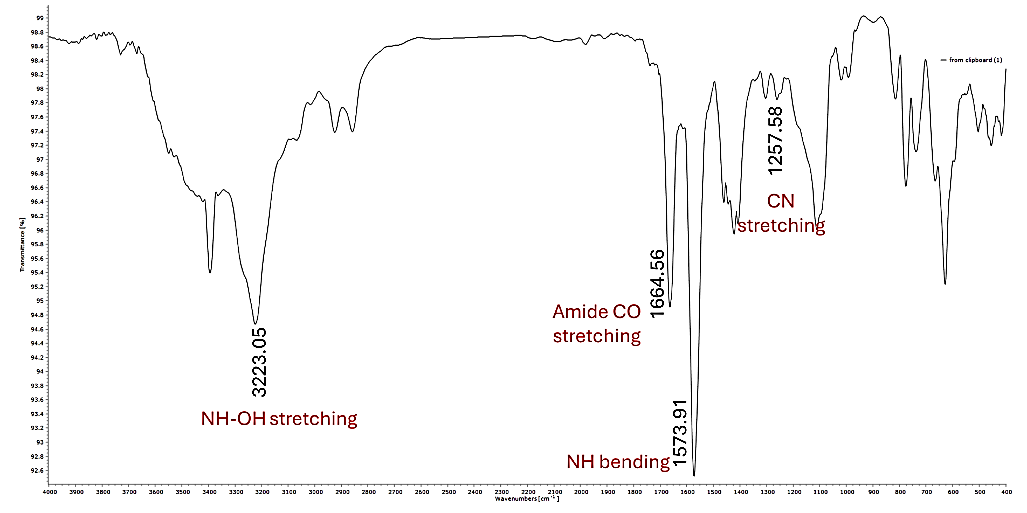 |

**Scheme 2:**


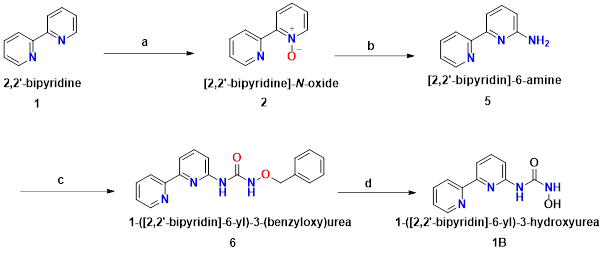


| **^1^H NMR spectrum of compound 5** |
| --- |
| 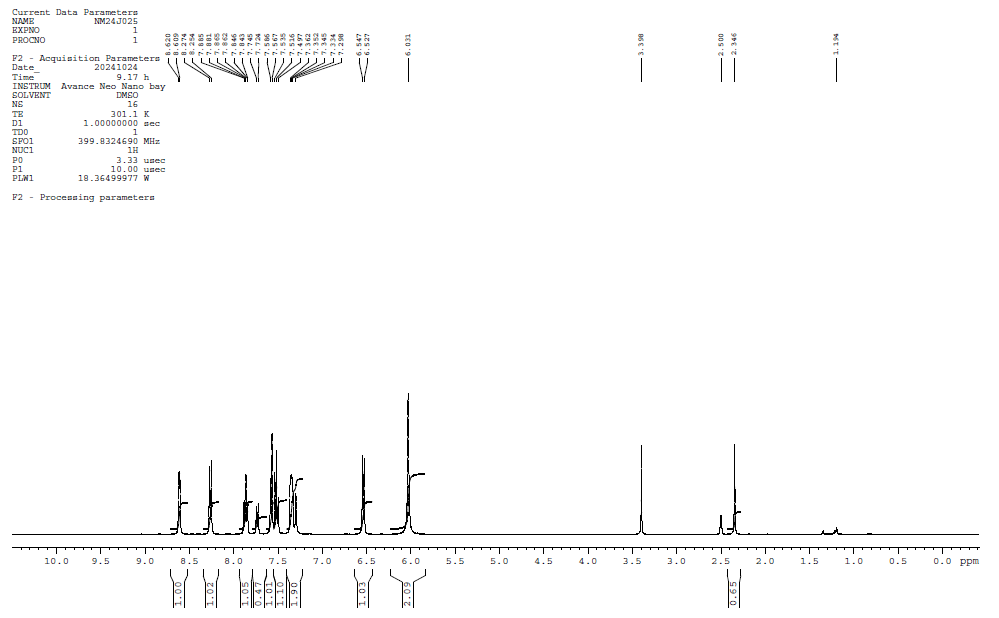 |
| **^13^C NMR spectrum of compound 5** |
| 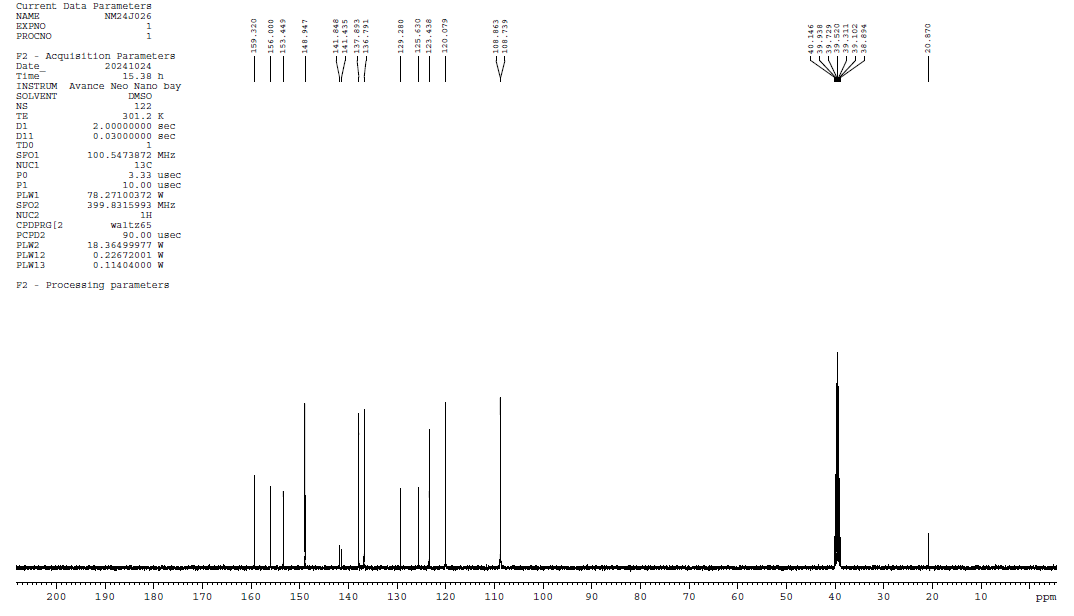 |
| **Mass spectrum of compound 5** |
| 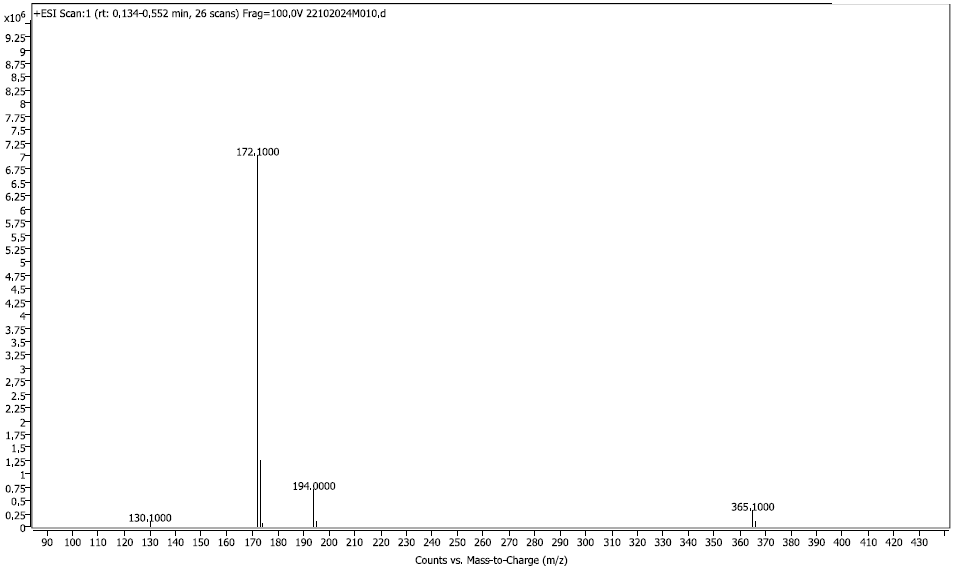 |
| **HPLC chromatogram of compound 5** |
|  |
| **^1^H NMR spectrum of compound 6** |
| 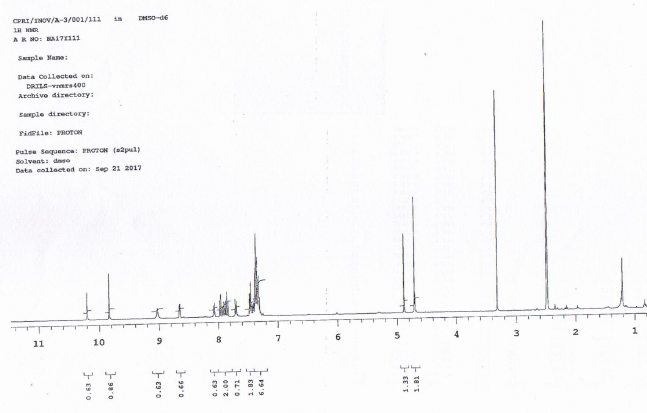 |
| **Mass spectrum of compound 6** |
|  |
| **^1^H NMR spectrum of compound 1B** |
| **^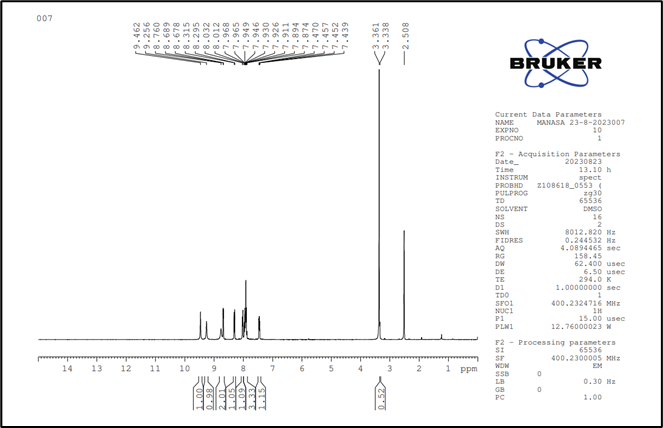^** |
| **^13^C NMR spectrum of compound 1B** |
| **^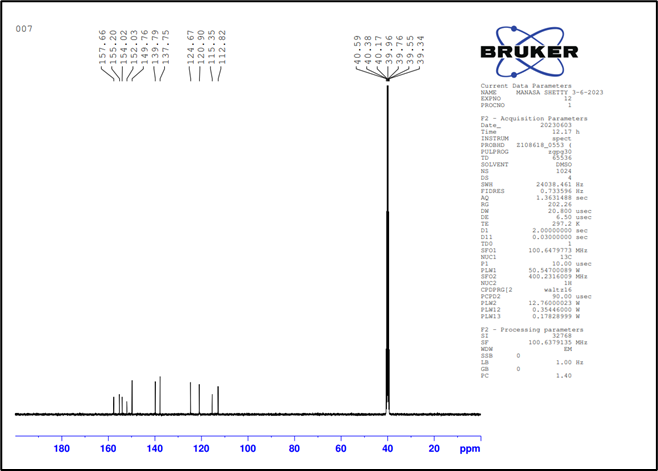^** |
| **Mass spectrum of compound 1B** |
| 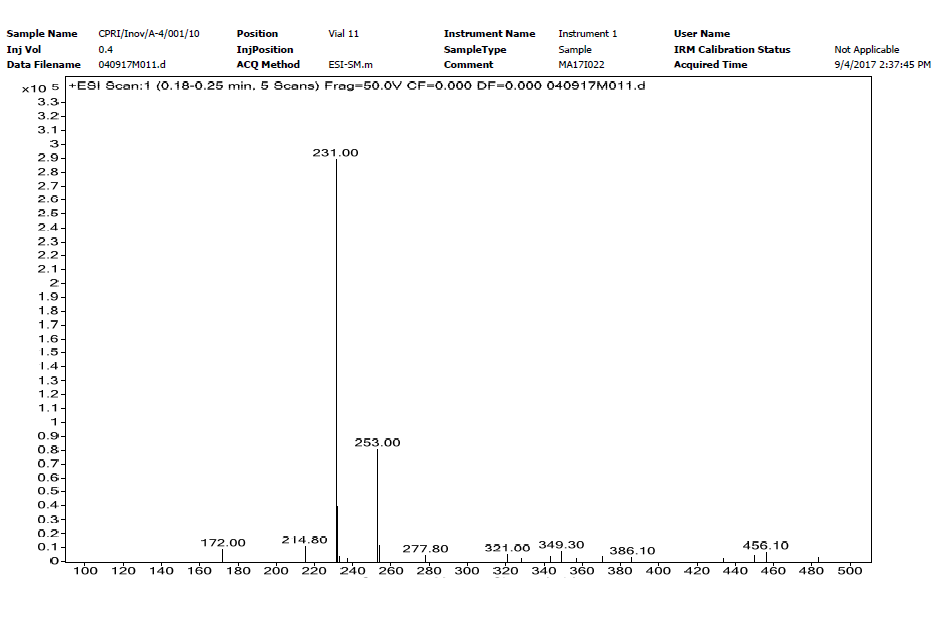 |
| **HPLC chromatogram of compound 1B** |
| 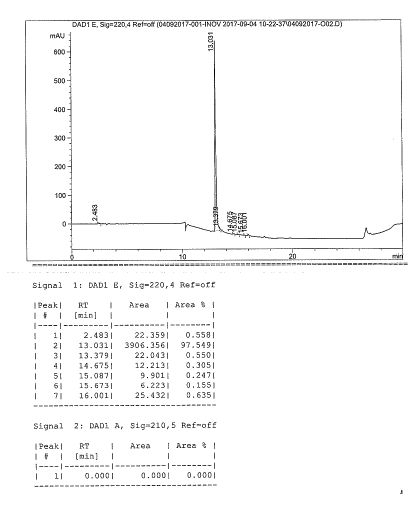 |
| **FTIR of compound 1B** |
| 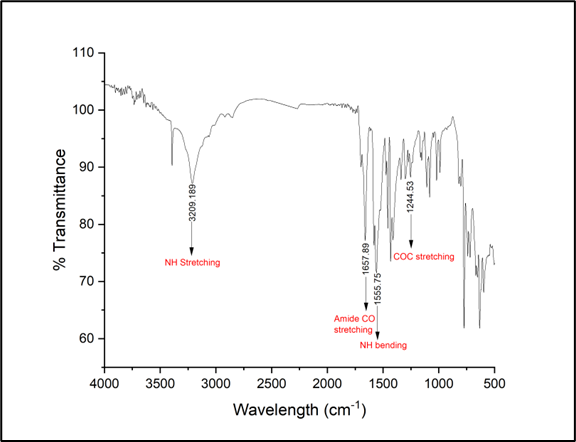 |
